# Notch-mediated Ephrin signaling disrupts islet architecture and **β** cell function

**DOI:** 10.1172/jci.insight.157694

**Published:** 2022-03-22

**Authors:** Alberto Bartolomé, Nina Suda, Junjie Yu, Changyu Zhu, Jinsook Son, Hongxu Ding, Andrea Califano, Domenico Accili, Utpal B. Pajvani

**Affiliations:** 1 Department of Medicine, Columbia University Irving Medical Center, New York, New York, USA.; 2 Department of Cancer Biology and Genetics, Sloan Kettering Institute, Memorial Sloan Kettering Cancer Center, New York, New York, USA.; 3 Systems Biology, Vagelos College of Physicians & Surgeons, Columbia University, New York, New York, USA.

**Keywords:** Endocrinology, Cell migration/adhesion, Diabetes, Islet cells

## Abstract

Altered islet architecture is associated with β cell dysfunction and type 2 diabetes (T2D) progression, but molecular effectors of islet spatial organization remain mostly unknown. Although Notch signaling is known to regulate pancreatic development, we observed “reactivated” β cell Notch activity in obese mouse models. To test the repercussions and reversibility of Notch effects, we generated doxycycline-dependent, β cell–specific Notch gain-of-function mice. As predicted, we found that Notch activation in postnatal β cells impaired glucose-stimulated insulin secretion and glucose intolerance, but we observed a surprising remnant glucose intolerance after doxycycline withdrawal and cessation of Notch activity, associated with a marked disruption of normal islet architecture. Transcriptomic screening of Notch-active islets revealed increased Ephrin signaling. Commensurately, exposure to Ephrin ligands increased β cell repulsion and impaired murine and human pseudoislet formation. Consistent with our mouse data, Notch and Ephrin signaling were increased in metabolically inflexible β cells in patients with T2D. These studies suggest that β cell Notch/Ephrin signaling can permanently alter islet architecture during a morphogenetic window in early life.

## Introduction

Pancreatic islets are composed of different hormone-secreting cells and a complex supporting niche that includes vasculature, nerves, and other cell types. Islets have distinct architectural patterns. Murine islets display a core composed primarily of β cells, with other endocrine cell types in the mantle. Islet architecture and morphology show important species-specific variations (1), but in all species, islet architecture is not random, reflecting a common mantle core organization of α and β cells when integrated with other islet components (2–4).

β Cells have differential functional capacity, as defined by electrophysiologic and insulin secretion parameters, when isolated, aggregated with other β cells, or in native islets (5, 6). Even within a single islet, β cells are highly heterogeneous in terms of electrical activity and insulin secretion, with specialized hubs orchestrating responses to glucose (7, 8). Whether these differences are affected by specific molecular guides that direct the architectural conformation of the islet, and whether these are altered in pathophysiology and contribute to disease progression, are not known. These questions are not easy to solve, since alterations in architecture are typically accompanied and, in mouse models, may in fact be caused by underlying β cell death or loss of maturity/identity (9–13). Nevertheless, altered islet architecture has long been associated with islet dysfunction in rodent models and human type 2 diabetes (T2D) (14–17).

Notch signaling, an evolutionarily conserved pathway critical for pancreas development and endocrine specification (18, 19), is “reactivated” in β cells from obese mice (20). Dynamic Notch activity alters the maturity status of the cell and contributes to obesity-induced β cell dysfunction (20). Whether these effects are permanent was not known. Using a potentially novel, doxycycline-dependent Notch gain-of-function model, we find that Notch-induced loss of cell maturity is reversible, but glucose intolerance persists, associated with disrupted and persistent islet architectural changes. Using a variety of methods in mouse and human islets, we identify a potentially novel Notch/Ephrin axis that affects β cell repulsion that determines islet architecture.

## Results

### Notch-induced β cell functional impairment.

**Figure 1:**
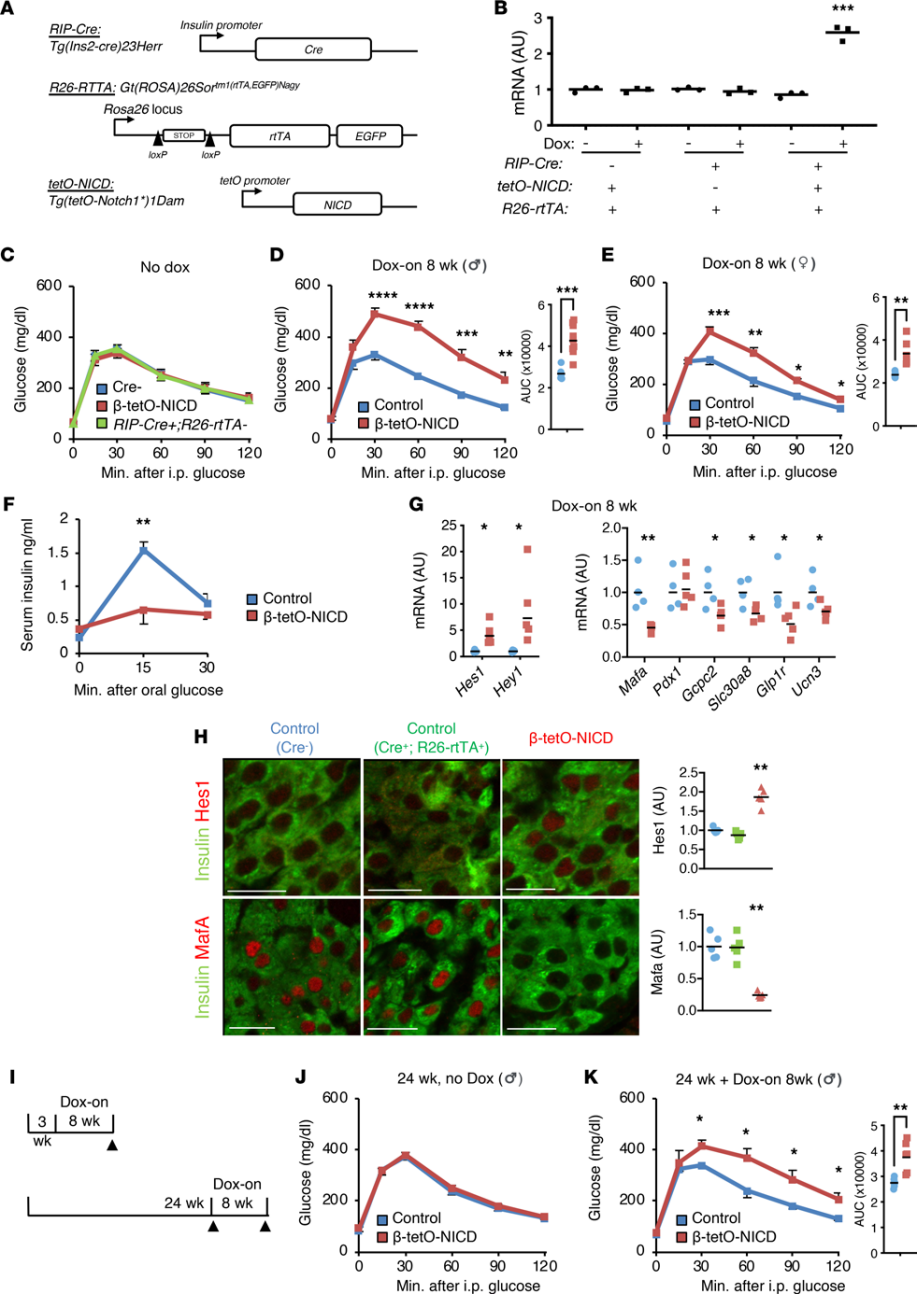
β Cell Notch activation induces glucose intolerance and loss of β cell maturity. (**A**) Generation of β-tetO-NICD mice. (**B**) *Hes1* expression in islets that were cultured overnight in the presence or absence of 1 μg/mL doxycycline (Dox). *n* = 3 mice per genotype. (**C**) Glucose tolerance test (GTT) in 8-week-old *Cre^–^*, β-tetO-NICD, and *Cre^+^ Rosa26-rtTA^–^* mice, prior to Dox exposure (*n* = 6–7 mice/group). AUC, area under the curve (mg × dL/min). (**D**) GTT in male and (**E**) female β-tetO-NICD and *Cre*^–^ controls after 8 weeks’ Dox (*n* = 7–9 mice/group). (**F**) Serum insulin after oral glucose challenge in male β-tetO-NICD and *Cre*^–^ controls, after 8 weeks’ Dox (*n* = 5 mice/group). (**G**) Gene expression in islets isolated from β-tetO-NICD and *Cre^–^* mice after 8 weeks’ Dox. Notch targets (left) and β cell maturity genes (right) (*n* = 4–5 mice/group). (**H**) Representative images of pancreatic sections from *Cre^–^* and *Cre^+^ Rosa26-rtTA^+^ tetO-NICD^–^* control and β-tetO-NICD mice after 8 weeks’ Dox, with antibodies directed against Hes1 (top) or Mafa (bottom) and insulin, with quantitation of β cell nuclear Hes1 and Mafa fluorescence intensity (*n* = 5 mice/group). Scale bars: 20 μm. (**I**) Experimental schematic used in Dox-on at weaning (top) or in older mice (bottom). (**J** and **K**) GTT in 24-week-old control and β-tetO-NICD males prior to (**J**) or after (**K**) Dox (*n* = 5–6 mice/group). All data are shown with group means ± SEM; *, *P* < 0.05, **, *P* < 0.01, ***, *P* < 0.001, ****, *P* < 0.0001 by 2-tailed *t* test.

We have previously shown that constitutive β cell Notch activity impairs β cell maturity and functional capacity (20). To determine whether these effects are specific to select developmental windows, we generated β cell–specific, doxycycline-inducible (Dox-inducible) Notch gain-of-function (β-tetO-NICD) mice (Figure 1A). As expected, Notch activity (as assessed by expression of the canonical Notch target *Hes1*) was specifically increased in islets that express all the necessary transgenes and in the presence of Dox (Figure 1B). Consistently, in vivo, in the absence of Dox, mice showed normal glucose tolerance (Figure 1C). With Dox exposure, both male and female β-tetO-NICD mice developed profound glucose intolerance (Figure 1, D and E), accompanied by impaired glucose-stimulated insulin secretion (GSIS) (Figure 1F). Islets from β-tetO-NICD mice showed increased expression of Notch targets and reduced markers of β cell functional maturity (Figure 1G), as well as increased β cell Hes1 with commensurate reduction of the maturity marker Mafa in fixed pancreata (Figure 1H), consistent with our previous observations (20). We observed a similar impairment in glucose tolerance even when Dox was started in older (24-week) mice (Figure 1, I–K). These data suggest that Notch activity is detrimental to β cell function even well past any developmental window.

### Notch-induced β cell functional impairment is not fully reversible.

**Figure 2:**
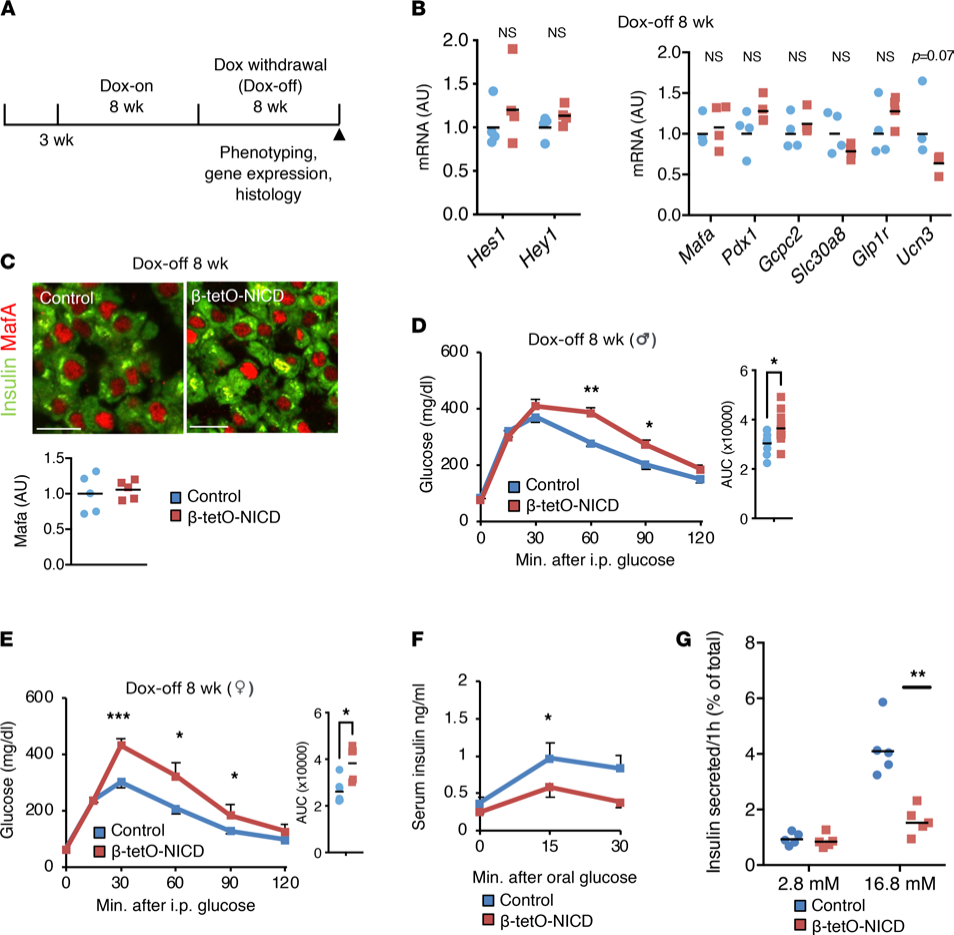
Notch-induced glucose intolerance is only partially reversible. (**A**) Experimental schematic used for Dox withdrawal (Dox-off). (**B**) Notch target (left) and β cell maturity gene (right) expression in islets isolated from 8 weeks’ Dox-off β-tetO-NICD and *Cre^–^* mice (*n* = 4 mice/group). (**C**) Representative images of pancreatic sections from 8 weeks’ Dox-off β-tetO-NICD and *Cre^–^* mice, with quantitation of β cell nuclear Mafa fluorescence intensity (*n* = 5 mice/group). Scale bars: 20 μm. (**D**) GTT in male and (**E**) female Dox-off β-tetO-NICD and *Cre^–^* controls (*n* = 7–9 mice/group). AUC, area under the curve (mg × dL/min). (**F**) Serum insulin after oral glucose challenge in Dox-off β-tetO-NICD and *Cre^–^* controls (*n* = 5 mice/group). (**G**) Glucose-stimulated insulin secretion (GSIS) in islets isolated from Dox-off β-tetO-NICD and *Cre^–^* mice, adjusted for islet insulin content (*n* = 5 mice/group). All data are shown with group means; *, *P* < 0.05, **, *P* < 0.01, ***, *P* < 0.001 by 2-tailed *t* test.

We next addressed the question of whether Notch-induced β cell functional defects were reversible (Figure 2A). Surprisingly, despite nearly indistinguishable expression of Notch targets and β cell maturity markers 4 weeks after Dox withdrawal (Supplemental Figure 1A; supplemental material available online with this article; https://doi.org/10.1172/jci.insight.157694DS1), β-tetO-NICD mice were still glucose intolerant (Supplemental Figure 1, B and C). Eight weeks after Dox withdrawal (Dox-off), Notch activity was at baseline and markers of β cell maturity had recovered (Figure 2, B and C), but both male and female β-tetO-NICD mice remained glucose intolerant relative to controls (Figure 2, D and E). Differences in glucose tolerance were further exacerbated by high-fat diet feeding, despite unchanged body weight (Supplemental Figure 1, D and E). Impaired glucose tolerance was associated with impaired insulin secretion after oral glucose challenge (Figure 2F). These effects are likely cell autonomous, as isolated islets from Dox-off β-tetO-NICD mice showed similar defects in in vitro GSIS (Figure 2G). These data indicate that β cell Notch exposure early in life leads to persistent impairments in β cell function, even after the Notch signal is removed.

### Disrupted islet cytoarchitecture and persistent GSIS impairment in β-tetO-NICD mice.

**Figure 3:**
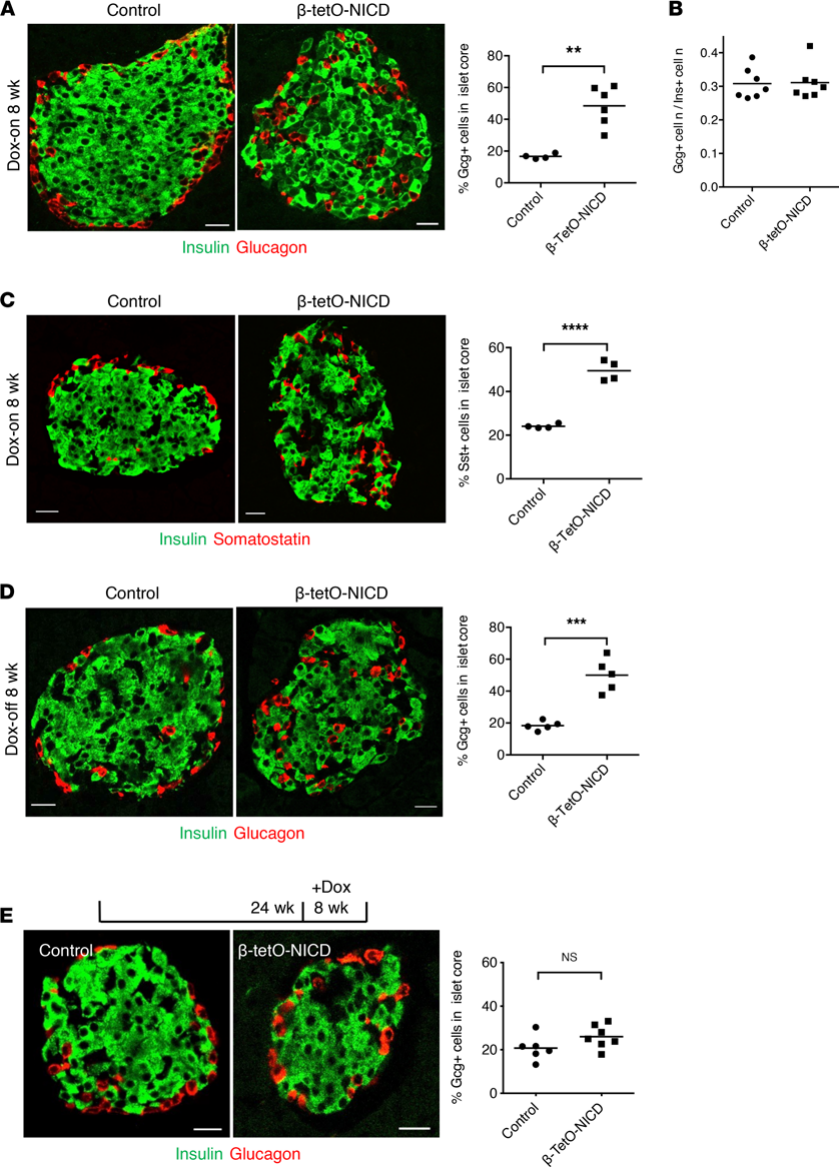
Disrupted islet cytoarchitecture in β-tetO-NICD mice. (**A**) Representative images of pancreatic sections from β-tetO-NICD and *Cre^–^* controls after 8 weeks’ Dox, with quantification of the percentage of glucagon^+^ (Gcg+) cells in the islet core (*n* = 4–6 mice/group). (**B**) Ratio of Gcg^+^ to insulin^+^ (Ins+) cells in β-tetO-NICD and Cre^–^ controls after 8 weeks’ Dox (*n* = 7 mice/group). (**C**) Representative images of pancreatic sections from β-tetO-NICD and *Cre^–^* controls after 8 weeks’ Dox, with quantification of the percentage of Somatostatin^+^ (Sst+) cells in the islet core (*n* = 4 mice/group). (**D**) Representative images of pancreatic sections from Dox-off β-tetO-NICD and *Cre^–^* controls, with quantification of Gcg^+^ cells in the islet core (*n* = 5 mice/group). (**E**) Representative images of pancreatic sections with quantification of Gcg^+^ cells in the islet core in 24-week-old β-tetO-NICD and *Cre^–^* controls after 8 weeks’ Dox (*n* = 6–7 mice/group). Scale bars: 20 μm. All data are shown with group means; **, *P* < 0.01, ***, *P* < 0.001, ****, *P* < 0.0001 by 2-tailed *t* test.

At sacrifice, we noted a markedly altered cytoarchitecture in β-tetO-NICD mice, with a 2.5× higher percentage of glucagon-positive α cells in the islet core (Figure 3A). We also found altered islet architecture in an independent β cell–specific Notch gain-of-function model (β-NICD; ref. 20), in which NICD is expressed in β cells perinatally (Supplemental Figure 2A). This finding suggested that altered architecture was not an artifact due to Dox. Similarly, *Cre^+^ rtTA^+^* mice lacking NICD showed normal mantle-core distribution of α cells (Supplemental Figure 2B). β to α cell ratio was unchanged between controls and β-tetO-NICD mice, suggesting that altered islet architecture was not due to loss or proliferation of either cell type (Figure 3B). Further, EGFP-mediated lineage tracing showed no evidence of β to α transdifferentiation (Supplemental Figure 2C). Importantly, we observed the same altered core-mantle distribution for δ cells in β-tetO-NICD mice (Figure 3C), suggesting a primary defect in Notch-active β cells that leads to disrupted localization of all endocrine cells of the islet.

Intriguingly, Dox withdrawal did not result in normalization of this ratio, with a near-identical increase in α cells in the islet core (Figure 3D), as seen in mice with continued Notch exposure. These data suggested a permanent alteration in islet architecture with early β cell Notch exposure but also led us to use this model to query whether there was a developmental window necessary for islet patterning. As shown earlier, delayed Notch exposure still gave rise to glucose intolerance (Figure 1K). But in this mouse model, we observed no differences in islet architecture (Figure 3E). This result suggests that the effects of Notch on islet architecture are age dependent and are probably more detrimental during the periods associated with islet morphogenesis and remodeling, such as perinatally or during early life.

### Altered Ephrin signaling in Notch gain-of-function islets.

We next surveyed Notch gain-of-function islets for potential explanations of altered cytoarchitecture based on existing literature. Cell adhesion molecules (CAMs) have been implicated in endocrine cell aggregation by enhancing cell-to-cell contact. For example, cadherin 1 (Cdh1) governs β cell homotypic interactions and their connectivity (21–24). A different CAM, epithelial CAM (Epcam), affects epithelial morphogenesis (25), and transgenic overexpression alters islet architecture with a high proportion of α and δ cells in the islet core (26). Epcam has also been described as a downstream effector of Notch signaling in hepatocellular carcinoma (27). But both Cdh1 and Epcam were unchanged in β-tetO-NICD islets (Supplemental Figure 3, A and B). Nonendocrine islet cells are also important determinants of islet architecture. Increased islet vascularization by β cell–derived VEGFA disrupts islet architecture (28–30). Macrophages affect islet morphogenesis, as well as islet remodeling after injury (29, 31). Again, however, β-tetO-NICD mice showed unchanged levels and distribution of islet endothelial cells and macrophages (Supplemental Figure 3, C and D). Similarly, other islet morphogenesis effectors such as Hnf1a (13), integrin β1/α5 (32), Ncam (10), and Slit-Robo signaling components (33) were unchanged in Notch-active islets (Supplemental Figure 4).

**Figure 4:**
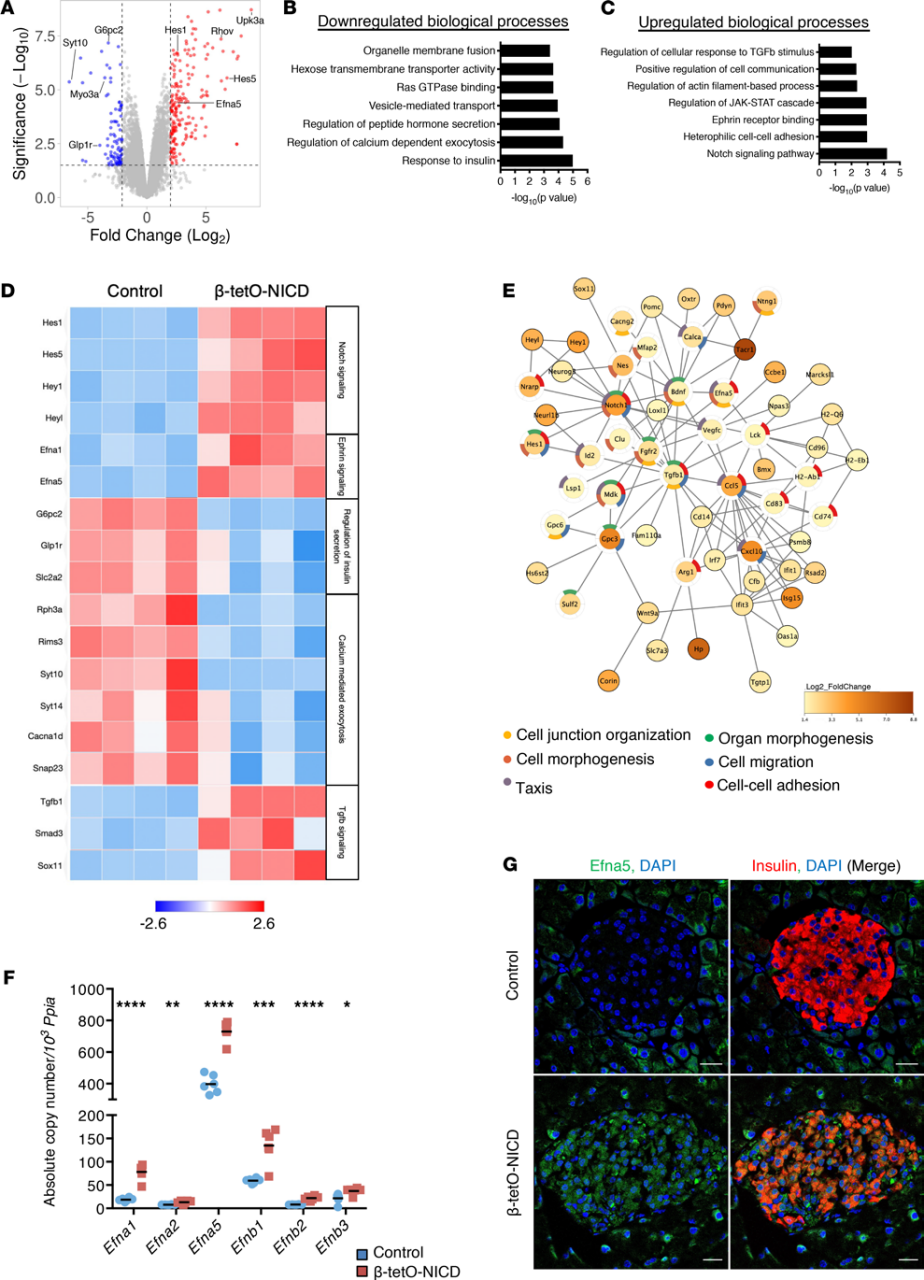
RNA-Seq of β-tetO-NICD islets reveals potential morphogenetic effectors. (**A**) Volcano plot showing differentially expressed genes (DEGs, adjusted *P* < 0.05) in islets from β-tetO-NICD and Cre^+^ controls after 8 weeks’ Dox. (**B**) Downregulated and (**C**) upregulated biological processes in β-tetO-NICD islets, after Gene Ontology enrichment analysis using Enrichr. (**D**) Heatmap of representative DEGs across samples, with log_2_-normalized gene expression value shown. Efna5, Ephrin A5. (**E**) STRING network analysis of upregulated DEGs in β-tetO-NICD islets showing enrichment analysis of processes associated with the network, highlighting processes associated with tissue morphogenesis. (**F**) Absolute quantitation, relative to invariant control *Ppia*, of genes encoding Ephrin ligands in islets isolated from β-tetO-NICD and control mice after 8 weeks’ Dox (*n* = 5–6 mice/group). (**G**) Representative images of pancreatic sections stained with antibodies against Efna5 and insulin from β-tetO-NICD and Cre^–^ controls after 8 weeks’ Dox. Scale bars: 20 μm. All data are shown with group means; *, *P* < 0.05, **, *P* < 0.01, ***, *P* < 0.001, ****, *P* < 0.0001 by 2-tailed *t* test.

As these targeted analyses were unrevealing, we performed RNA-Seq in islets isolated from controls and β-tetO-NICD. Consistent with previous results, gene set enrichment analysis (GSEA) of Notch-active islets did not match islets of mice lacking Robo1/Robo2 (34) (Supplemental Figure 5A). But commensurate with impaired GSIS, β-tetO-NICD islets showed downregulation of genes and processes associated with normal β cell function, as well as some intriguing upregulated candidate effectors for altered islet architecture, including signaling pathways associated with tissue morphogenesis and remodeling (Figure 4, A–D). To narrow down this list, we conducted STRING analyses to build association networks between differentially expressed genes. Among the downregulated genes, we found networks enriched in altered secretory function but not with tissue morphogenesis (Supplemental Figure 5B). On the contrary, Gene Ontology enrichment of upregulated biological processes was more revealing, with 2 hubs of potential interest: TGF-β and Ephrin signaling (Figure 4E). Transgenic *Tgfb1* expression in the β cell causes fibrosis and altered islet architecture (35), consistent with Notch effects (36–38), but fibrosis was unchanged in Notch-active islets (Supplemental Figure 6). Ephrin receptors and ligands are expressed in islets and β cell lines, where they can affect β and α cell function (39, 40). β-tetO-NICD islets showed a parallel upregulation of all expressed ligands, with *Efna5* as the most abundantly expressed (Figure 4F). We also observed increased Efna5 staining in β-tetO-NICD islets (Figure 4G), consistent with a direct effect of Notch.

### Efna5 inhibits islet reaggregation and β cell adhesion.

**Figure 5:**
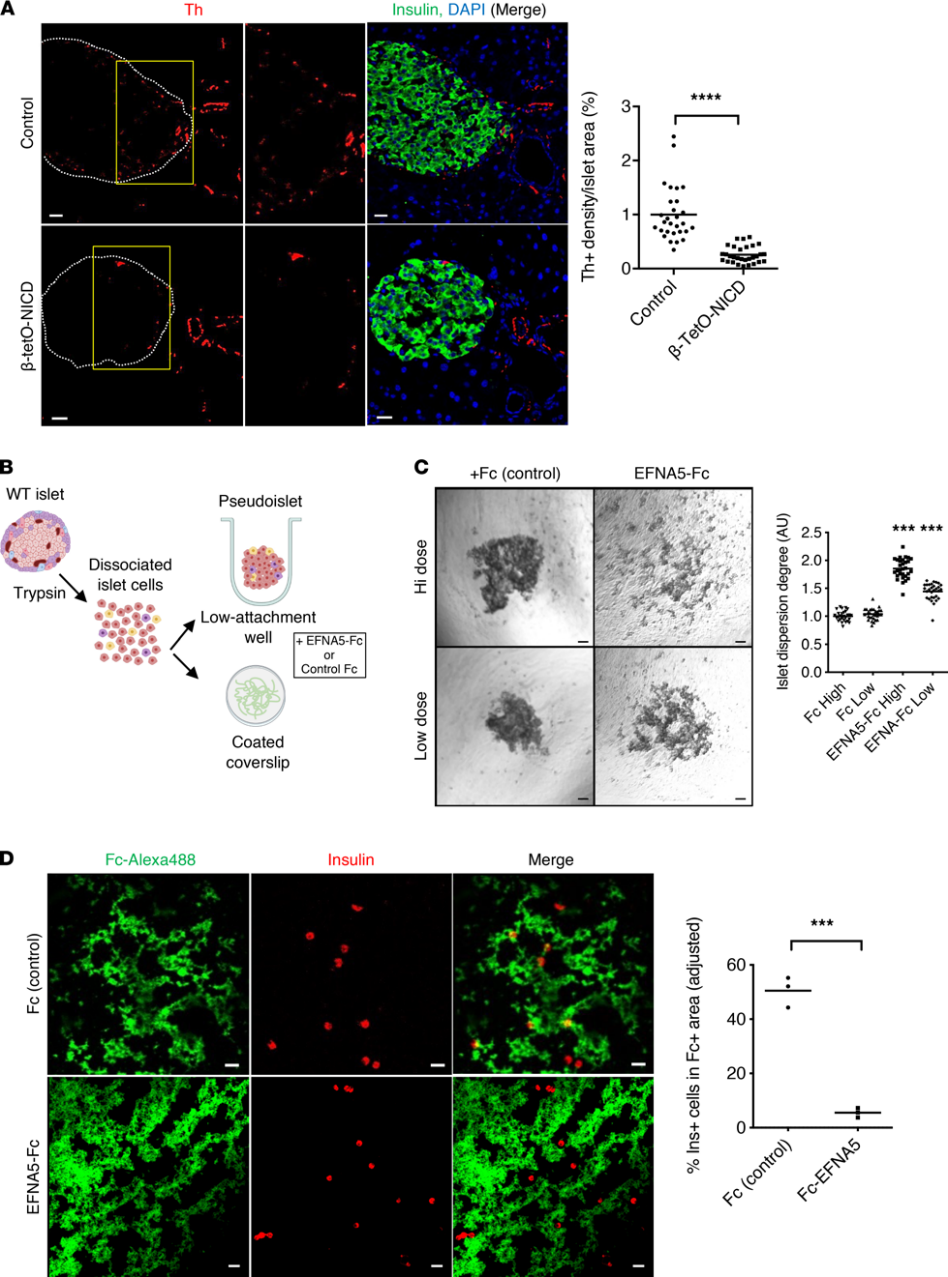
Repulsive effects of EFNA5 on mouse β cells. (**A**) Representative images and morphometric analysis of Th-positive area in β-tetO-NICD and *Cre*^–^ controls after 8 weeks’ Dox. Individual islets from at least 5 mice/group are plotted. (**B**) Experimental workflow for pseudoislet formation and cell repulsion assays. Islets were dissociated into single cells that were incubated in ultra-low-attachment wells for pseudoislet formation, or applied to coated coverslips for adhesion assays, in the presence of EFNA5-Fc chimera or Fc control. (**C**) Representative images and quantitation of pseudoislet formation of dispersed islet cells after 4-day exposure to EFNA5-Fc or Fc control, at high (55 nM) or low (13.75 mM) dose. Data pooled from 3 independent experiments. (**D**) Representative images and quantitation of β cells in EFNA5-Fc–coated condition (normalized to Fc control). Data shown as average of 3 independent experiments. Scale bars: 20 μm. All data are shown with group means; ***, *P* < 0.001, ****, *P* < 0.0001 by 2-tailed *t* test.

We next tested repercussions of higher Efna5 in Notch-active islets. One of the best-characterized functions of Ephrin signaling is repulsion of migrating axons (41–43). Sympathetic innervation of the developing pancreas occurs soon after endocrine progenitor specification and is linked to islet maturation (44). Depletion of sympathetic fibers during pancreas development results in altered islet morphogenesis, with more α cells in the islet core (45). Thus, we quantified the tyrosine hydroxylase–positive (Th-positive) area in islets of control and β-tetO-NICD mice and found a depletion of sympathetic fibers in Notch-active islets (Figure 5A). Eight weeks after Dox withdrawal, islet sympathetic innervation was restored to normal (Supplemental Figure 7A), consistent with normalization of expression of Ephrin ligands (Supplemental Figure 7B). As a parallel test, we embedded dispersed islet cells from β-NICD and control islets in a Matrigel disk surrounding a dorsal root ganglia (DRG) explant (45). After several days, sympathetic neurite outgrowth from DRG reached the disk containing dispersed β cells but less so with Notch-active β cells (Supplemental Figure 8). These data support the effects of Notch in repulsion of sympathetic fibers, potentially mediated by Ephrin signaling.

**Figure 6:**
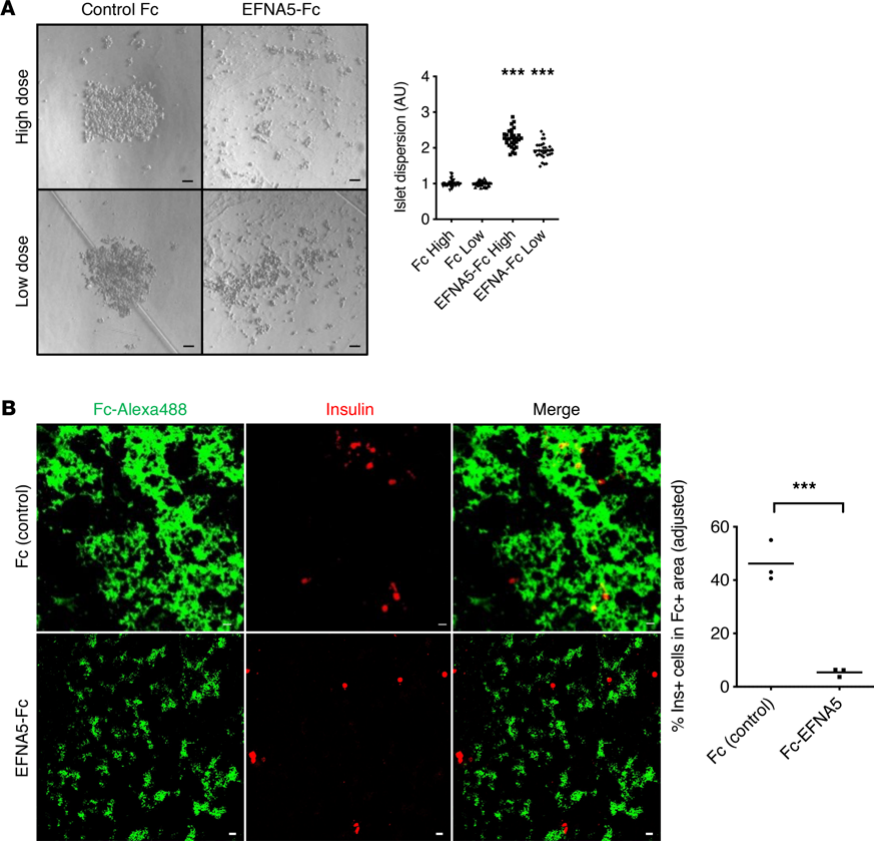
Repulsive effects of EFNA5 in human islets. (**A**) Representative images and quantitation of pseudoislet formation of dispersed cells from human nondiabetic donor islets, after 4-day exposure to EFNA5-Fc or Fc control, at high (55 nM) or low (13.75 mM) dose. Data pooled from 3 independent experiments. (**B**) Representative images and quantitation of human β cells in EFNA5-Fc–coated condition (normalized to Fc control). Data shown as average of 3 independent experiments. Scale bars: 20 μm. All data are shown with group means; ***, *P* < 0.001 by 2-tailed *t* test.

We next assayed effects of Efna5 on islet morphogenesis using primary dispersed islet cells (Figure 5B). Ephrin ligands are membrane bound and activate cognate receptors in neighboring cells (Ephrin forward signaling). Soluble Ephrin ligands are inactive but can be activated with forced clustering with antibodies (46). We conjugated EFNA5-Fc (or Fc control) with anti-Fc IgG, then treated dispersed islet cells cultured in ultra-low-attachment wells to form “pseudoislet” aggregates that resemble the native islets (47). EFNA5-Fc did not change cell viability by trypan blue dye exclusion (not shown), but we observed a marked, dose-dependent reduction in pseudoislet formation with EFNA5-Fc (Figure 5C). The same concentrations of unconjugated EFNA5-Fc had no impact on pseudoislet formation (not shown). We also probed the effect of TGF-β in the same experimental paradigm and found no effect on pseudoislet formation (Supplemental Figure 8). We next assayed the adhesion of of dispersed β cells to surfaces coated with either clustered Fc or EFNA5-Fc. Random coating patterns were generated, which were visualized by the use of a fluorescence-conjugated antibody. While β cells showed no preference for coated/uncoated Fc regions, they strikingly avoided EFNA5-Fc (Figure 5D), which we quantitated using 2 image analysis algorithms (Supplemental Figure 10). Importantly, EFNA5 similarly impaired pseudoislet formation from dispersed donor human islet cells (Figure 6A). Likewise, human β cells avoided regions coated with EFNA5-Fc (Figure 6B).

### Increased EFNA5 expression in islets from human donors with type 2 diabetes.

**Figure 7:**
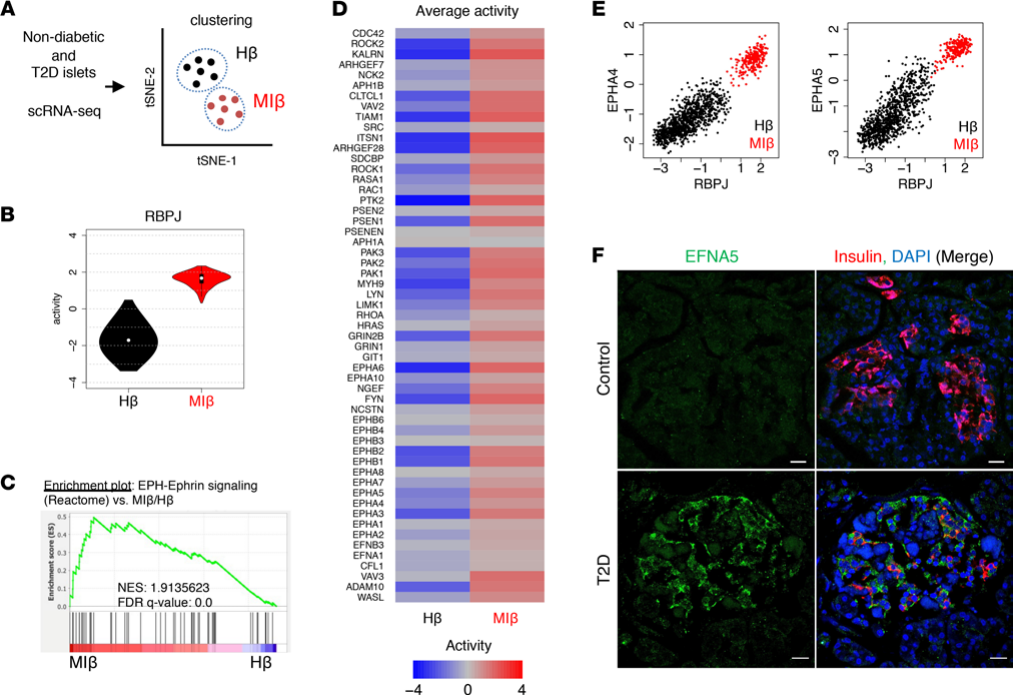
Increased Notch and Ephrin signaling in metabolically inflexible human β cells. (**A**) scRNA-Seq of islets from nondiabetic and T2D patients, with β cells subclustered by metabolic pathways to define healthy (Hβ) and metabolically inflexible (MIβ) as per a previous report (48). tSNE, t-distributed stochastic neighbor embedding. (**B**) Violin plots of RBPJ activity in Hβ and MIβ cell populations. (**C**) GSEA of EPH-Ephrin signaling in DEGs of MIβ versus Hβ populations. NES, normalized enrichment score. (**D**) Average activity of EPH-Ephrin signaling components in Hβ and MIβ populations. (**E**) RBPJ activity as compared with EPH-Ephrin signaling components, with each dot representing a single Hβ (black) and MIβ (red) cell. Extended figure with all EPH-Ephrin signaling components and Pearson’s *r* values in Supplemental Figure 11A. (**F**) Representative images of pancreatic sections stained with antibodies against EFNA5 and insulin from nondiabetic and T2D patients. Extended figure with additional staining in Supplemental Figure 12B. Scale bars: 20 μm.

These data suggested that altered Notch-induced Efna5 can affect islet morphogenesis, in both mouse and human islets. To interrogate endogenous Notch and Ephrin signaling in human β cells, we used single-cell RNA-Seq (scRNA-Seq) data obtained from nondiabetic and T2D islet donors (48). We transformed mRNA expression data into a protein activity matrix to build the islet-specific regulatory network (48), then inferred Notch and Ephrin protein activities using metaVIPER (49) in healthy β cells compared with T2D β cells. Using iterClust (50), we defined nondiabetic enriched β cells with strong INS/MAFA activity but no metabolic inflexibility markers as “healthy β cells” (Hβ cells) and T2D enriched β cells with increased α cell and metabolic inflexibility markers as “metabolically inflexible β cells” (MIβ cells) (Figure 7A and ref. 48). In MIβ cells, we observed higher Notch activity, as defined by the protein activity of its nuclear effector, recombination signal binding protein for immunoglobulin kappa J region (RBPJ), as compared with the Hβ population (Figure 7B). GSEA of Ephrin signaling components revealed a similar enrichment in MIβ as compared to Hβ cells (Figure 7C), leading to a higher EPH-Ephrin activity score (Figure 7D). We next plotted RBPJ activity and expression of Ephrin signaling components at the single-cell level, which showed striking and highly significant positive correlations (Figure 7E and Supplemental Figure 11A). Consistent with data from murine Notch-active islets, *EFNA5* was the most increased EPH-Ephrin–related gene in the MIβ population (Supplemental Figure 11B). We confirmed these observations by staining EFNA5 in the pancreata of organ donors with and without T2D. While EFNA5 expression was barely detected in nondiabetic controls, islets from patients with T2D showed altered islet morphology (Supplemental Figure 12A), as well as a striking increase in EFNA5 in β and non-β islet cells (Figure 7F and Supplemental Figure 12B).

## Discussion

Islets of Langerhans are architecturally patterned organs, embedded within the exocrine pancreas, with which they share common developmental origins. Islets have a unique, nonrandom, spatial configuration and cellular composition that partly differ between species (1–4). Processes governing cellular sorting within the islet are not clear, including how cell fate decisions are coordinated within the physical mechanisms that organize different cell types to form a complex 3D structure.

Our findings provide an important link between Notch, a master regulator of cell fate, and the architectural configuration of the islet. These studies benefited from the ability to dynamically activate (and inactivate) Notch activity at will, which allowed us to disentangle changes in islet architecture with other cellular processes (51–53). For example, islet architecture defects can often be traced to cell death or loss of identity (9–13). We observed normalization of β cell maturity when Notch activity was returned to baseline, in agreement with the capacity of β cells to recover their mature functional status after resolution of the provoking insult (54, 55). Islet architecture, however, remained disrupted, suggesting that islet ultrastructure may be less reparable. As Dox-off β-tetO-NICD mice showed impaired GSIS and glucose intolerance, we tentatively conclude that islet architecture is critical for β cell function, consistent with the well-established notion of the impact of β cell connectivity, companions, and niche for β cell electrical activity and secretory function (5, 6) and architectural defects seen in rodent models (14, 15), and in human T2D (16, 17). Nevertheless, despite apparent normalization of Notch activity, we cannot fully exclude the possibility that remnant Notch activity remains.

We considered the possibility that islet architecture defects are due to Notch-induced changes in cell maturity. However, we observed impaired β cell function regardless of when Notch activity was induced, whereas islet architecture defects were only observed when activation was induced perinatally or at weaning. To this latter point, since most pancreatic endocrine cells in mice are formed during the first 2 months of life (56), and these have a very long life span (57), we speculate that changes in Notch activity can alter islet architecture during this “morphogenetic window,” with potential lifelong consequences. This leads to the attractive hypothesis that *NOTCH2* polymorphisms associated with T2D in genome-wide association studies may have primary impact at this time, or even earlier during pancreas development (58, 59). Future studies are needed to test this possibility, as well as whether excess Notch activity may prove detrimental, by means of islet architecture disruption, in response to physiologic (pregnancy) or pathologic (obesity, pancreatitis) islet stressors.

We also observed lower sympathetic innervation in the Notch-active islet. Pancreas sympathetic innervation occurs after endocrine specification (45), a step dependent on derepression of Ngn3 by Notch (18). In the absence of sympathetic signals, embryonic pancreata from *Th^–/–^* embryos show elevated Notch signaling and impaired endocrine differentiation (60). This might reflect the existence of a feedback loop, in which sympathetic cues in the developing pancreas curtail Notch activity that in turn allow for further sympathetic innervation. In our model, normal innervation was recovered after normalization of the Notch signal. This highlights the plasticity of islet innervation and fits with current knowledge about dynamics of the process, as transplanted islets can be fully reinnervated within weeks (61).

Notch-Ephrin crosstalk is well documented (62) but primarily in developmental contexts (63–65). Our data showed high expression of Ephrin ligands, in particular Efna5, in developed Notch-active islets, wherein it mediates a repulsive effect on β cells. These data are consistent with the positive correlation between Notch activity and Ephrin signaling components in human β cells at the single-cell level, with a highly marked increase in the MIβ cell. Ephrin signaling has been previously characterized as an important pathway for islet cell communication. EphA receptor forward signaling inhibits insulin secretion, while Ephrin ligand reverse signaling has a stimulatory effect (39). A similar finding was reported for α cells, where EphA forward signaling is able to inhibit basal glucagon secretion (40), dependent on Ephrin ligand expression by β cells (66). This context makes increased EFNA5 in islets from patients with T2D islets very intriguing. We hypothesize that high EFNA5 may be an adaptive response to limit glucagon secretion, since islets from these patients show a markedly altered structure with a higher number of α cells. Although we find that EFNA5 impairs human pseudoislet formation, the contribution of ephrins from β and other islet cells to islet destructuring and secretory dysfunction in human T2D is not yet clear and will require further investigation. But, as inhibition of Ephrin receptors with the use of small chemicals has been shown to potentiate insulin secretion in both mouse and human islets (67), this may represent a novel pharmacologic strategy to ameliorate β cell dysfunction.

In sum, we describe a potentially novel role of Notch to disrupt islet architecture, but only when the Notch signal was applied to the islet morphogenetic window in early life, due to altered Ephrin signaling. While Notch effects on β cell maturity were reversible, repercussions on architecture persisted. We note the potential of Notch inhibition to improve islet function (20, 68), but this may prove problematic due to potential homeostatic effects in other tissues (69). Nevertheless, understanding how Notch/Ephrin signaling determines islet architecture may have broader therapeutic implications. For instance, stem cell–derived “islet-like” clusters may benefit from Ephrin signaling tuning to allow better cohesion or the integration of nonendocrine supporting cells. Of equal importance, our results may provide impetus for renewed investigation of mechanisms by which islet cytoarchitecture is determined and maintained, an important facet of β cell biology.

## Methods

### Animals.

*RIP-Cre: Tg(Ins2-cre)23Herr* (70); *Gt(ROSA)26Sor^tm1(rtTA,EGFP)Nagy^* (71); and Tg(tetO-Notch1*)1Dam (72) mouse lines were maintained on a mixed background (C57BL/6J, 129X1/SvJ, BALB/c). *R26-NICD: Gt(ROSA)26Sor^tm1(Notch1)Dam^* (18) mice were maintained on a C57BL/6J background. Mouse lines were obtained from The Jackson Laboratory, with the exception of Tg(tetO-Notch1*)1Dam, provided by Ben Stanger (University of Pennsylvania, Philadelphia, Pennsylvania, USA). Mice were weaned and maintained on standard chow (Purina Mills no. 5053), and 1 mg/mL doxycycline (Alfa Aesar) was provided in drinking water. Males and females were used for experiments. Islets from males were used for gene expression experiments. Islets from male and female mice were used without distinction for all ex vivo experiments.

### Antibodies and chemicals.

We used antibodies against insulin (IR002) and somatostatin (A0566) from Dako, Agilent; Acta2 (ab5694), Adgre1 (ab6640), GFP (ab6673), and Mafa (ab26405) from Abcam; Hes1 (sc-25392) and endomucin (sc-65495) from Santa Cruz Biotechnology; Cdh1 (#3195), glucagon (#2760), and vimentin (#5741) from Cell Signaling Technology; Epcam (g8.8) from Developmental Studies Hybridoma Bank; TH (AB152) from MilliporeSigma; desmin (RB-9014-P) from Thermo Fisher Scientific; and Efna5 (AF3743), recombinant human EFNA5-Fc chimera (374-EA), and recombinant mouse TGF-β1 (7666-MB) from R&D Systems, Bio-Techne. Human IgG Fc fragment (009-000-008) and anti–Fc–Alexa Fluor 488 (709-545-098) were from Jackson ImmunoResearch.

### Glucose tolerance tests and GSIS.

Glucose tolerance tests were performed after 2 g/kg body weight intraperitoneal injection. For in vivo GSIS, an oral glucose challenge of 3 g/kg body weight was used. Ex vivo GSIS on isolated islets was performed as previously described (20). Insulin was detected by ELISA (Mercodia).

### Immunostaining, confocal microscopy, and image analysis.

Pancreata fixed in 4% paraformaldehyde (PFA) were processed as previously described (20). Pancreas sections from humans used in this study were previously described (73). Confocal imaging was performed with an Axio Observer Z1 with LSM 710 scanning module (Zeiss). Imaging was performed in a single confocal microscopy session for each experiment. For each session, the photomultiplier voltage settings (below 600 V) and laser transmission (≤2%) for each fluorophore were determined to maximize the dynamic range of the signal. Controls were used for each experiment to confirm specific signals. All images were obtained in a 1024 × 1024 pixel format. For microscope operation and image gathering, ZEN (Zeiss) software was used. Fluorescence intensity of nuclear signals (Mafa/Hes1) was determined with the open-source Fiji software (https://imagej.net/software/fiji/), as previously described (20). Morphometric analyses were performed with Fiji using the thresholding function to measure areas.

### Islet isolation and culture and human islet studies.

Islets were isolated by collagenase P (Roche Applied Science) digestion of whole pancreata followed by Histopaque density gradient centrifugation as described (74). When required, islets were maintained ex vivo in islet media (5.5 mM glucose RPMI 1640 supplemented with 10% FBS and 1% penicillin-streptomycin, all from Gibco, Thermo Fisher Scientific). For islet dispersion experiments, isolated islets were incubated overnight and dissociated with trypsin (Gibco, Thermo Fisher Scientific). Dispersed cells were cultured in islet medium. Human islets from a single nondiabetic donor were obtained through the Integrated Islet Distribution Program. Islets were cultured in PIM(S) media supplemented with 5% human serum (Prodo). After arrival at our institution, the islets were cultured overnight, and 3 independent experiments were performed in the next 3 days using approximately 400 IEQ/experiment. For pseudoislet formation assays, dispersed mouse or human cells were seeded in ultra-low-attachment 96-well plates (Nunclon Sphera, Thermo Fisher Scientific) at a density of 2000 cells/well. After 4 days, images were taken with an Axiovert 25 inverted microscope (Zeiss) and analyzed to determine islet dispersion with the Spatial Statistics plugin (75) for Fiji on watershed binary images. The cumulative density function of the distances between each cell to any other was obtained for every image, and area under the curve was normalized and annotated as “dispersion degree.” Representative pictures shown for all experiments have quantified values approximated to the final average.

### Neurite–β cell contact assay.

We followed an experimental procedure previously described (45) but adapted to developed β cells. In brief, DRG from 4-week-old mice were harvested and incubated in ice-cold Hanks balanced salt solution (HBSS). Each dorsal root ganglion was embedded in a 2 μL Matrigel drop in the center of a collagen-coated well of a 4-well dish. Matrigel drops were allowed to polymerize at 37°C. In parallel, islets isolated from β-NICD or control mice were dispersed into single cells. Cell suspensions were diluted 1:1 with Matrigel and placed around the drop containing the DRG. After polymerization, wells were filled with islet medium supplemented with 100 ng/mL NGF (Thermo Fisher Scientific, 11050-HNAC), and medium was replaced every other day. After 7 days, cells were fixed with 4% PFA, permeabilized with 0.1% Triton X-100, and stained with antibodies against insulin and Tubb3. *Z*-stacks of confocal images were collected. Only the region reached by outgrowing neurites was considered for quantification, and the percentage of β cells contacting neurites was determined. 3D reconstructions were obtained with Imaris Viewer.

### RNA-Seq, differential gene expression, and enrichment analyses.

Library construction and RNA-Seq were performed by Macrogen. Islets from 4 controls (*Cre^+^ Rosa26-rtTA^+^ tetO-NICD^–^*) and 4 β-tetO-NICD (*Cre^+^ Rosa26-rtTA^+^ tetO-NICD^+^*) after 8 weeks of Dox exposure were used. Raw counts were pseudoaligned to the mouse transcriptome (GRCm38) by Kallisto (76) and gene count tables generated. Differential gene expression analyses were performed with *limma*, implemented in the BioJupies application (77). Enrichment analyses were performed with Enrichr (78). STRING analysis was performed using the open-source Cytoscape application (https://cytoscape.org). Networks built from differentially expressed genes were filtered by only considering nodes with high connection degree and immediate neighbors. Enrichment analyses were performed with these genes (79, 80). GSEA to compare β-Robo1/2dKO (34) and β-tetO-NICD mice was performed by using the GSEA-Preranked tool of the GSEA software (http://gsea-msigdb.org). The Ephrin gene set can be found at https://reactome.org/content/detail/R-HSA-3928664 (identifier R-HSA-3928664). Default parameters were used for running GSEA. For absolute quantitation of transcripts, PCR products were gel-purified and cloned into pCR4-TOPO vectors (Thermo Fisher Scientific). A standard curve with defined number of copies per each gene was run in parallel to cDNA samples to determine copy number. *Ppia* was used as the invariant control.

### Regulatory networks and transcriptional regulator activity analysis.

Islet-specific regulatory networks were reverse-engineered by ARACNe (81) on an individual patient basis. ARACNe was run with 200 bootstrap iterations using 1813 transcription factors (genes annotated in Gene Ontology molecular function database, as GO:0003700, transcription factor activity; or as GO:0003677, DNA binding, and GO:0030528, transcription regulator activity; or as GO:00034677 and GO:0045449, regulation of transcription), 969 transcriptional cofactors (a manually curated list, not overlapping with the transcription factor list, built upon genes annotated as GO:0003712, transcription cofactor activity, or GO:0030528 or GO:0045449), and 3370 signaling pathway–related genes (annotated in GO Biological Process database as GO:0007165, signal transduction, and in GO cellular component database as GO:0005622, intracellular, or GO:0005886, plasma membrane). Parameters were set to 0 data processing inequality tolerance and mutual information *P* value (using mutual information computed by permuting the original data set as null model) threshold of 1 × 10^–^^8^. Protein activity profiles were then generated by metaVIPER by integrating across all 11 donor-specific regulatory networks (49). Dimension reduction and clustering analysis were performed as previously reported (48), focused on Hβ and MIβ cells, defined by clusters MI-1 and MI+2, respectively (48).

### Ephrin ligand clustering and β cell adhesion to EFNA5-coated surfaces.

Ligands were clustered as described (82). In brief, Fc or EFNA5-Fc was mixed at a 1:5 molar ratio with Alexa Fluor 488–anti–human Fc IgG, followed by 2 hours of gentle agitation at room temperature. Clusters were used in pseudoislet formation and cell adhesion assays. For cell adhesion, round coverslips were washed in KOH/ethanol (EtOH) solution, followed by functionalization in 5% (3-glycidyloxypropyl) trimethoxysilane in EtOH and 0.01% poly-l-ornithine coating (MilliporeSigma). After washing and air-dying the coverslips, 1.35 μM Fc or EFNA5-Fc cluster drops were applied to the surface and placed in the incubator for 2 hours. Coverslips were carefully washed with HBSS and further coated with laminin 50 μg/mL in HBSS for 1 hour. Dispersed mouse islet cells were seeded in complete islet medium, or in PIM(S) in the case of human islet cells. Cells were fixed after 96 hours, then stained with anti-insulin antibodies. The percentage of β cells colocalizing with Alexa Fluor 488–IgG was calculated and normalized to total Alexa Fluor 488^+^ area.

### Data availability.

RNA-Seq data of β-tetO-NICD islets have been deposited in National Center for Biotechnology Information’s Gene Expression Omnibus (GEO) database (GSE193888). scRNA-Seq data of human islets are also available in GEO under the accession number GSE98887.

### Statistics.

Results are shown as mean ± SEM. Differences between 2 groups were calculated using a 2-sided Student’s *t* test. Differences between multiple groups and a control were calculated by 1-way ANOVA followed by Dunnett’s multiple-comparison post hoc test. A *P* value of less than 0.05 was considered statistically significant.

### Study approval.

The Columbia University Institutional Animal Care and Use Committee approved all animal procedures. Human islets were harvested from deceased donors without any identifying information. Written informed consent and IRB approval were obtained at islet isolation centers.

## Author contributions

AB conducted experiments and contributed to the experimental design, discussion, and writing of the manuscript. NS, JY, and CZ conducted experiments and contributed to the review and editing of the manuscript. JS and HD performed the human islet experiments and analyzed the data. UBP, DA, and AC oversaw research and contributed to the experimental design, discussion, and writing of the manuscript.

## Supplementary Material

Supplemental data

## Version 1 02/15/2022

In-Press Preview

## Version 2 03/22/2022

Electronic publication
