# Supplementary material for: Notch-mediated Ephrin signaling disrupts islet architecture and β cell function
Source: JCI Insight. 2022 Mar 22;7(6):e157694. doi: 10.1172/jci.insight.157694 (PMC8986078; doi:10.1172/jci.insight.157694)
Supplement: Supplemental data [file jciinsight-7-157694-s045.pdf]

## Supplemental Figure S1

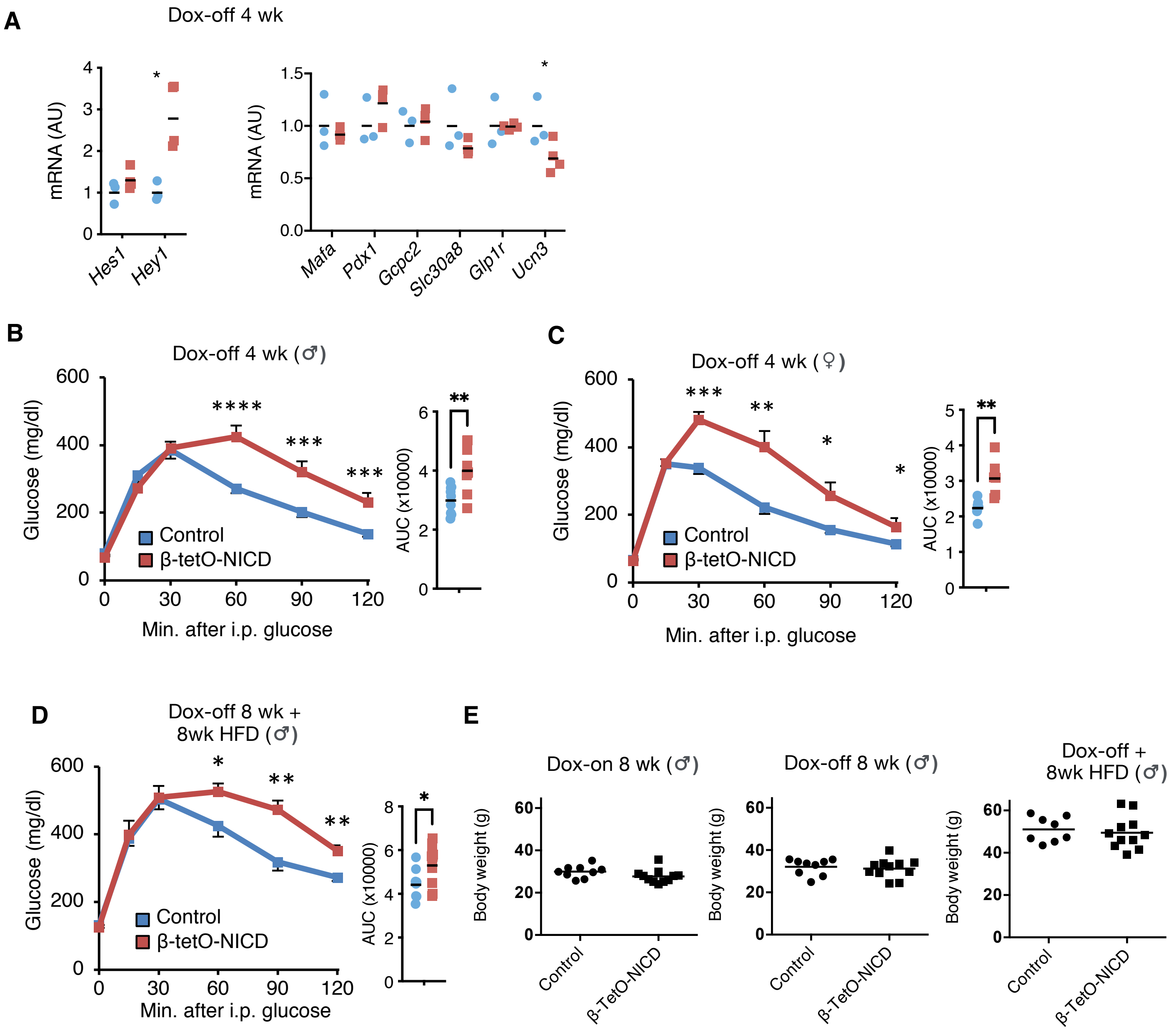


Supplemental Figure S1. Further characterization of β-tetO-NICD mice.
(A) Notch target (left) and β cell maturity (right) gene expression in islets isolated from β-tetO-NICD and $Cre^-$ mice after 4 weeks of Dox withdrawal (N=3-4 mice/group).
(B) GTT in males and (C) females after 4 weeks of Dox withdrawal. (N=7-9 mice/group). AUC: area under curve (mg × $dl^{-1}$ × min).
(D) GTT in Dox-off β-tetO-NICD and control male mice, followed by 8 weeks HFD (total 16 weeks Dox withdrawal, N=9-11 mice/group).
(E) Body weight in Dox 8 weeks (left), Dox-off 8 weeks (middle), 8 weeks Dox-off followed by 8 weeks HFD (right) male mice (N=9-11 mice/group).
All data are shown with group means; *, $P < 0.05$, **, $P < 0.01$, ***, $P < 0.001$, ****, $P < 0.0001$ by two-tailed *t* test.

## Supplemental Figure S2

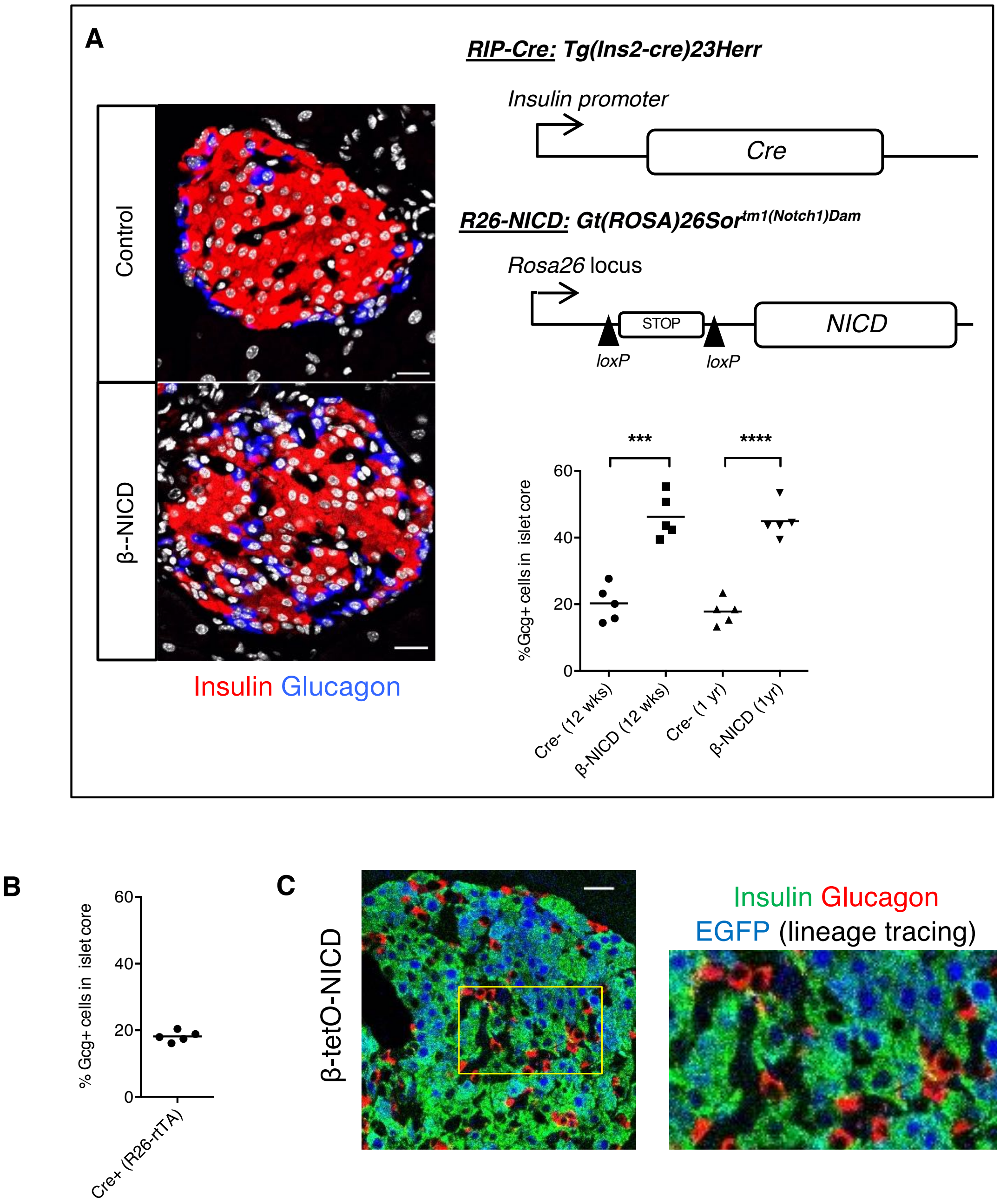


Supplemental Figure S2. Islet architectural abnormalities in Notch gain-of-function mice.
(A) Generation of β-NICD mice, and representative images of pancreatic sections from β-NICD and *Cre*$^-$ control mice, with quantification of Gcg+ cells in the islet core in 12 week and 1 year old mice (N=5 mice/group).
(B) Quantification of Gcg+ cells in the islet core of 8 week old *Cre*$^+$*; Rosa26-rtTA*$^+$*; tetO-NICD*$^-$ control mice.
(C) Representative image of pancreatic sections from Dox-off β-tetO-NICD mice. EGFP is expressed in all cells that underwent Cre-mediated recombination in the Rosa26 locus due to NICD-EGFP bicistronic expression). EGFP staining is only observed in Ins+ cells. Scale bars: 20 µm

## Supplemental Figure S3

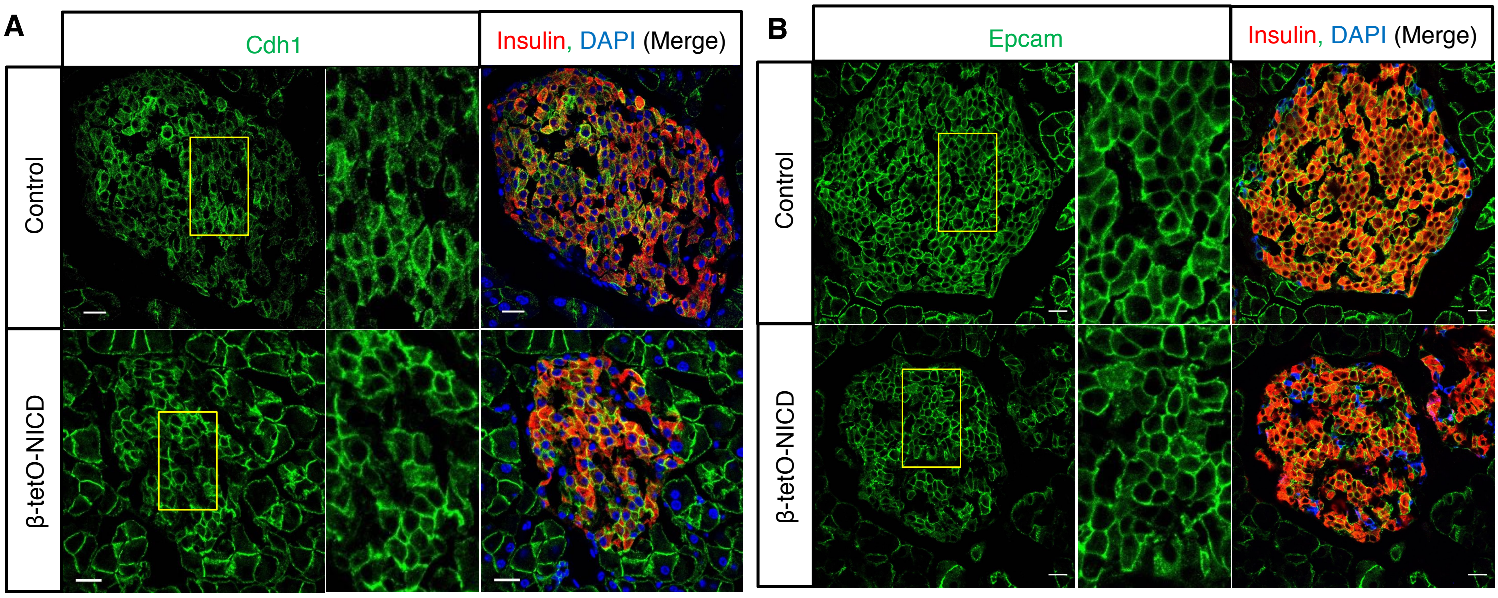


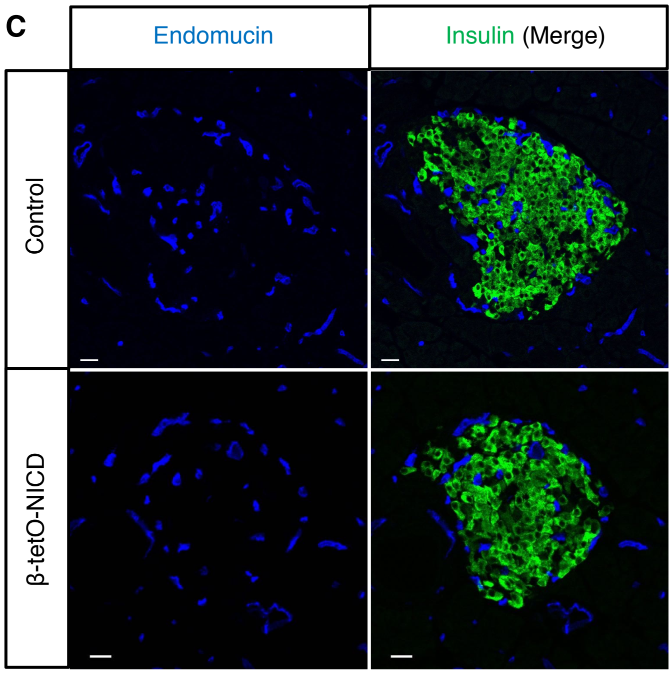


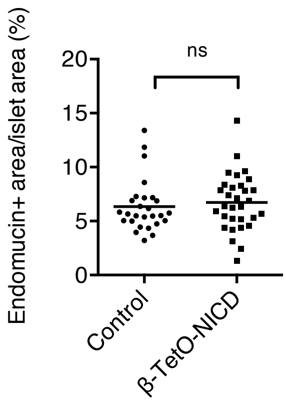


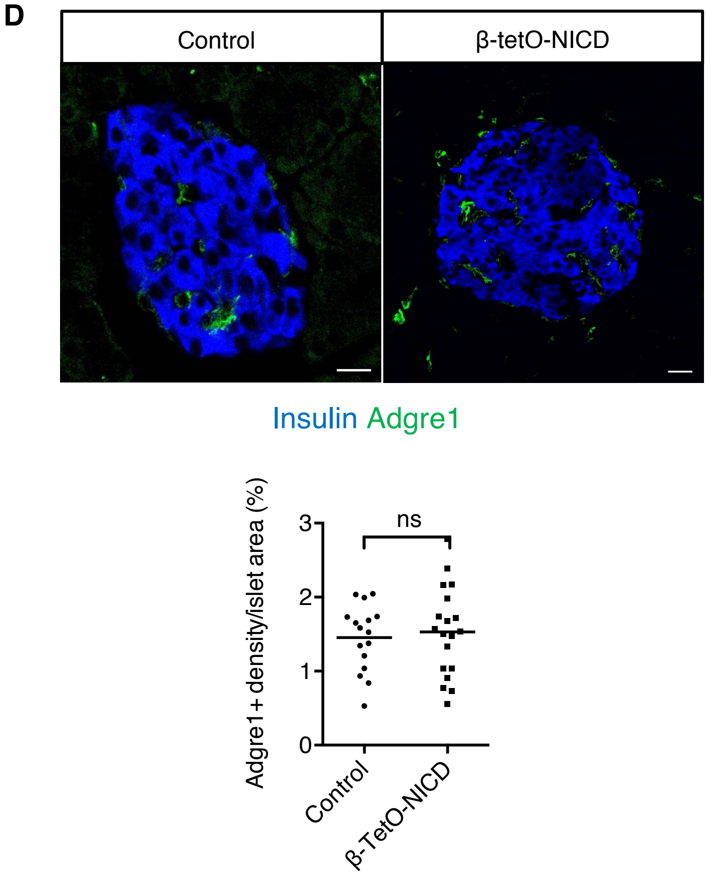


Supplemental Figure S3. Histological characterization of β-tetO-NICD mice.
(A) Representative images of pancreatic sections from β-tetO-NICD and Cre[-] controls after 8 weeks Dox stained with antibodies against Cdh1 and (B) Epcam.
(C) Representative images, and morphometric analysis of Endomucin+ endothelial cell and (D) Adgre+ macrophage area, normalized to total islet area in β-tetO-NICD and *Cre*[-] controls after 8 weeks Dox. Individual islets from at least 4 mice/group are plotted. Scale bars: 20 µm

**Supplemental Figure S4**

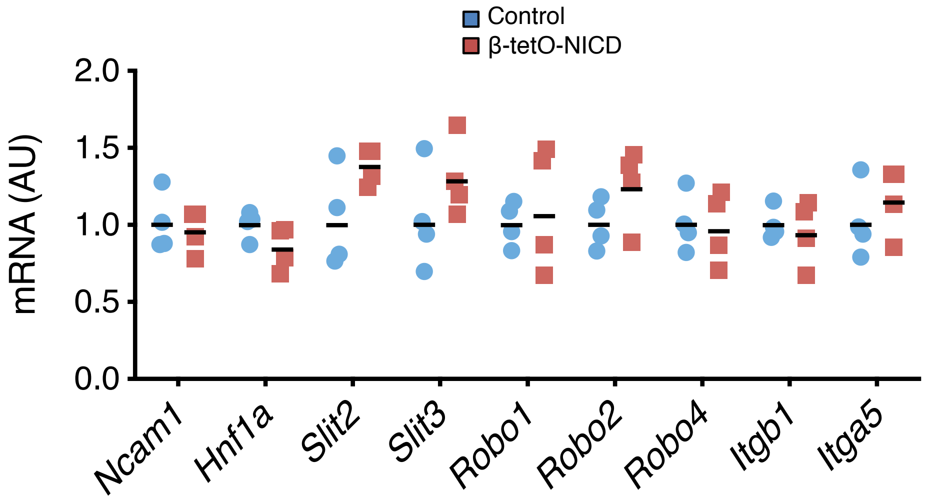


Supplemental Figure S4. Unchanged known morphogenetic pathways in Notch-active islets.

Gene expression of other described islet morphogenetic effectors in islets from β-tetO-NICD and *Cre*⁻ controls after 8 weeks Dox. N=4 mice/group. All data are shown with group means.

## Supplemental Figure S5

A

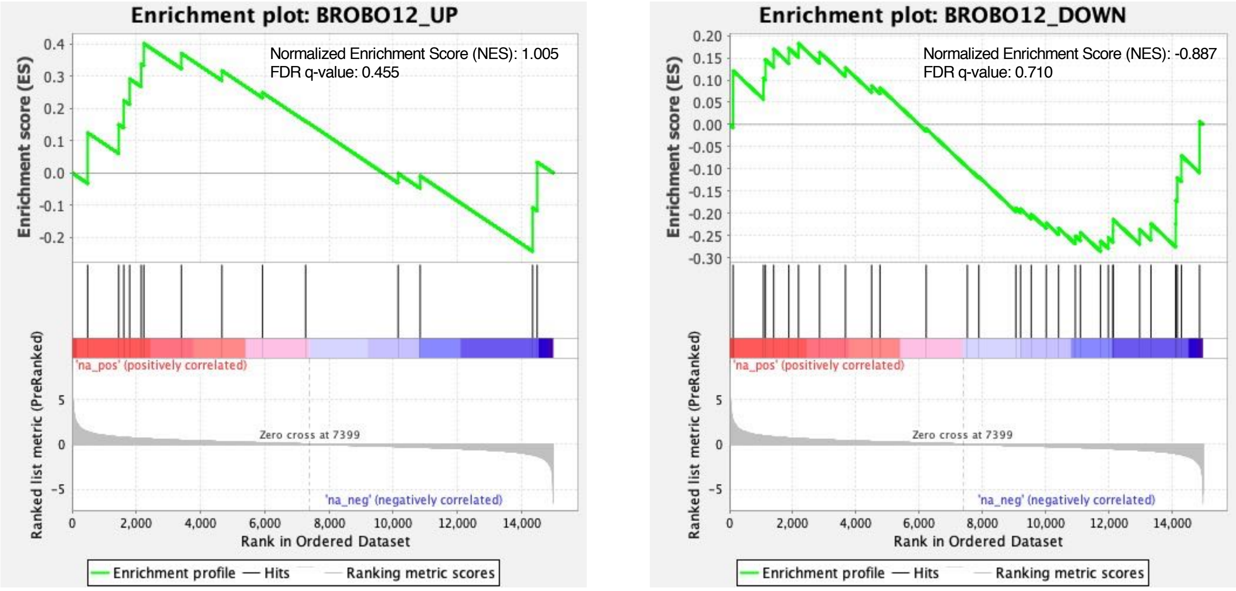


B

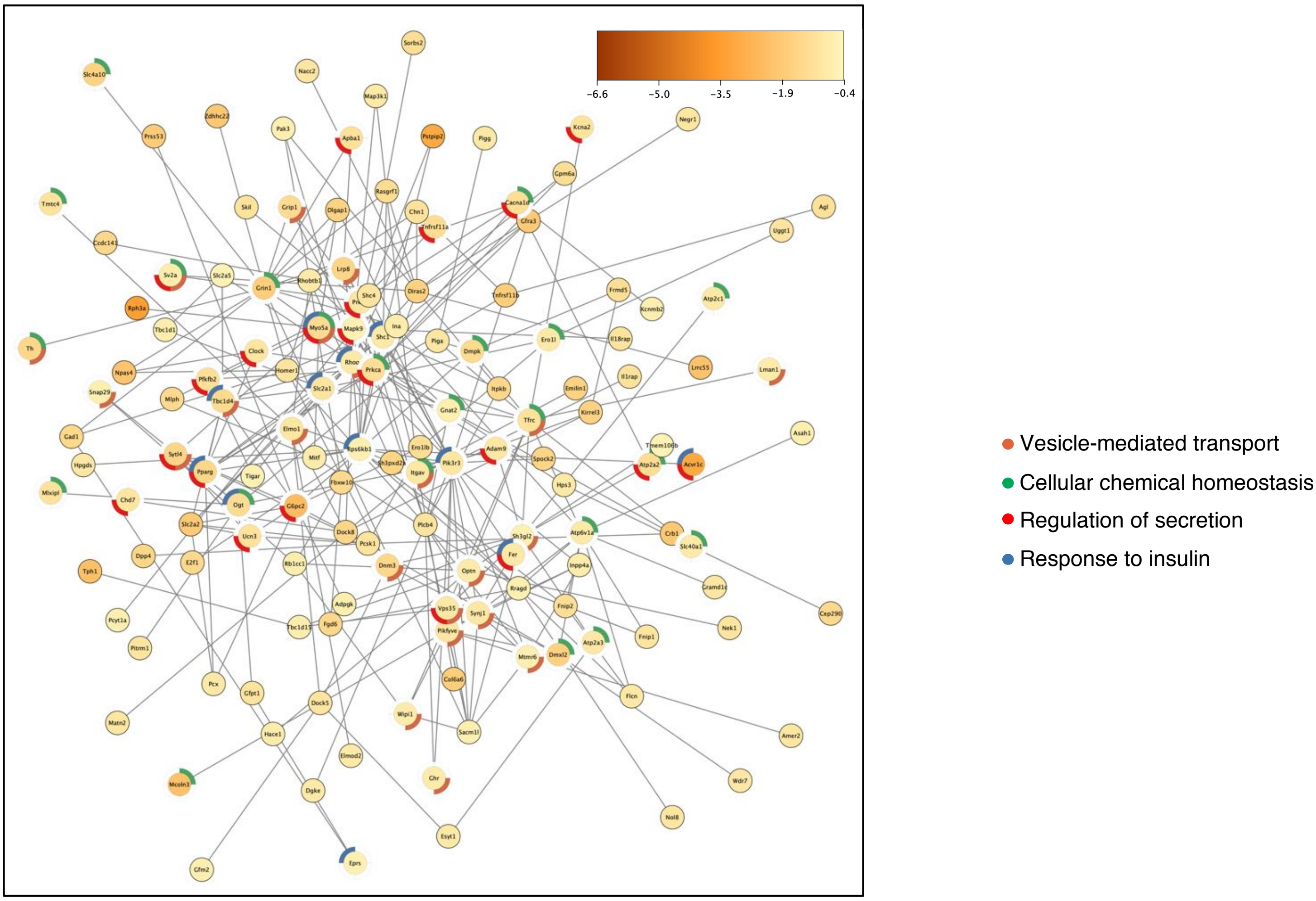


Supplemental Figure S5. RNA-seq characterization of Notch-active islets
(A) Gene set enrichment analysis, comparing upregulated (left) and downregulated (right) genes in Robo1/2 dKO β cells (Adams et al, 2021) with β-tetO-NICD islets.
(B) STRING network from downregulated DEGs in β-tetO-NICD islets. Enrichment analysis of processes associated with the network, highlighting processes associated with β cell secretory function.

**Supplemental Figure S6**

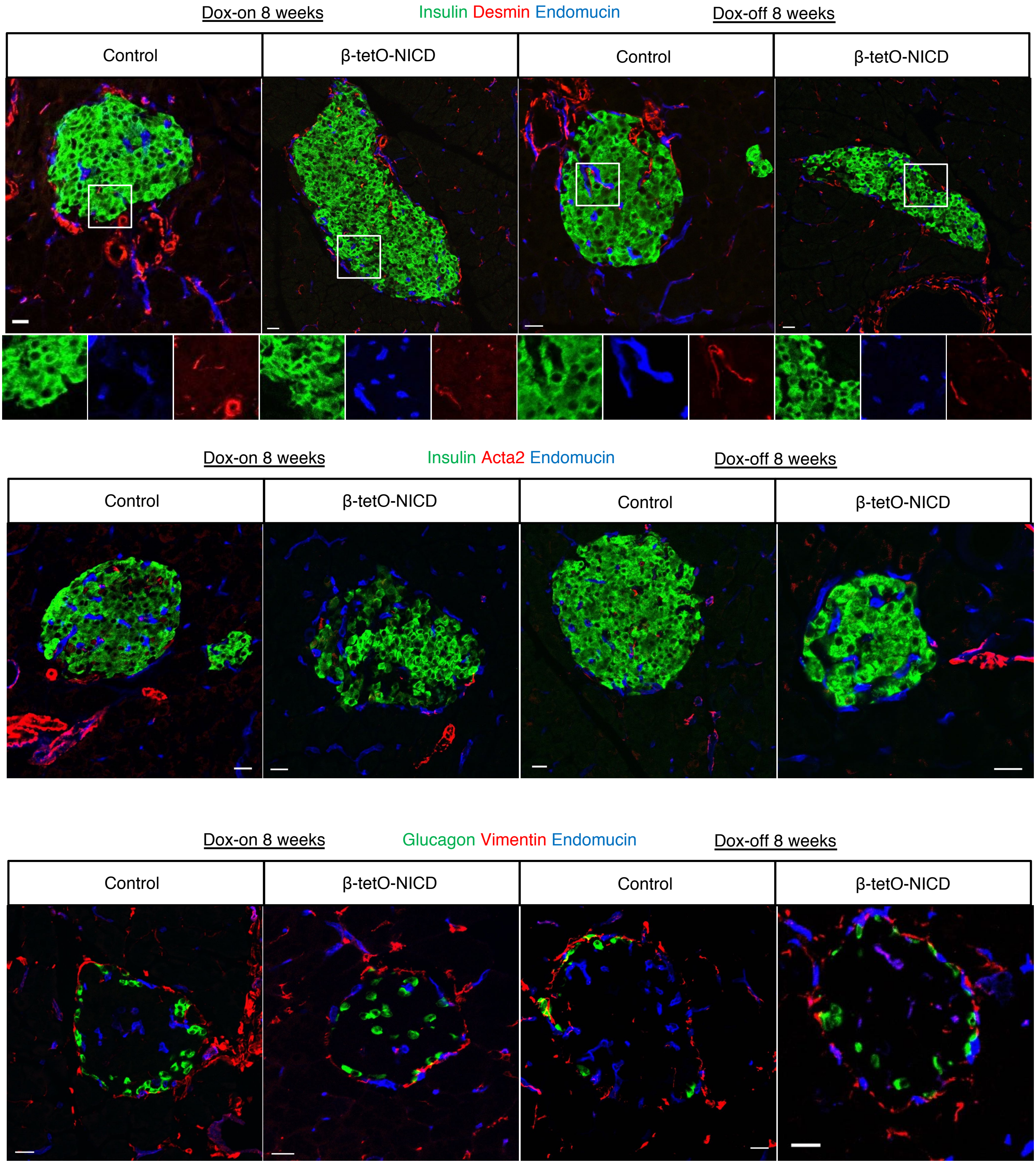


Supplemental Figure S6. Unchanged fibrosis markers in Notch-active islets.
Representative image of pancreatic sections from β-tetO-NICD and control mice, either after 8 weeks Dox or 8 weeks Dox-off, stained with fibrosis markers Desmin (top), Acta2 (middle) and Vimentin (bottom). Scale bars: 20 µm

## Supplemental Figure S7

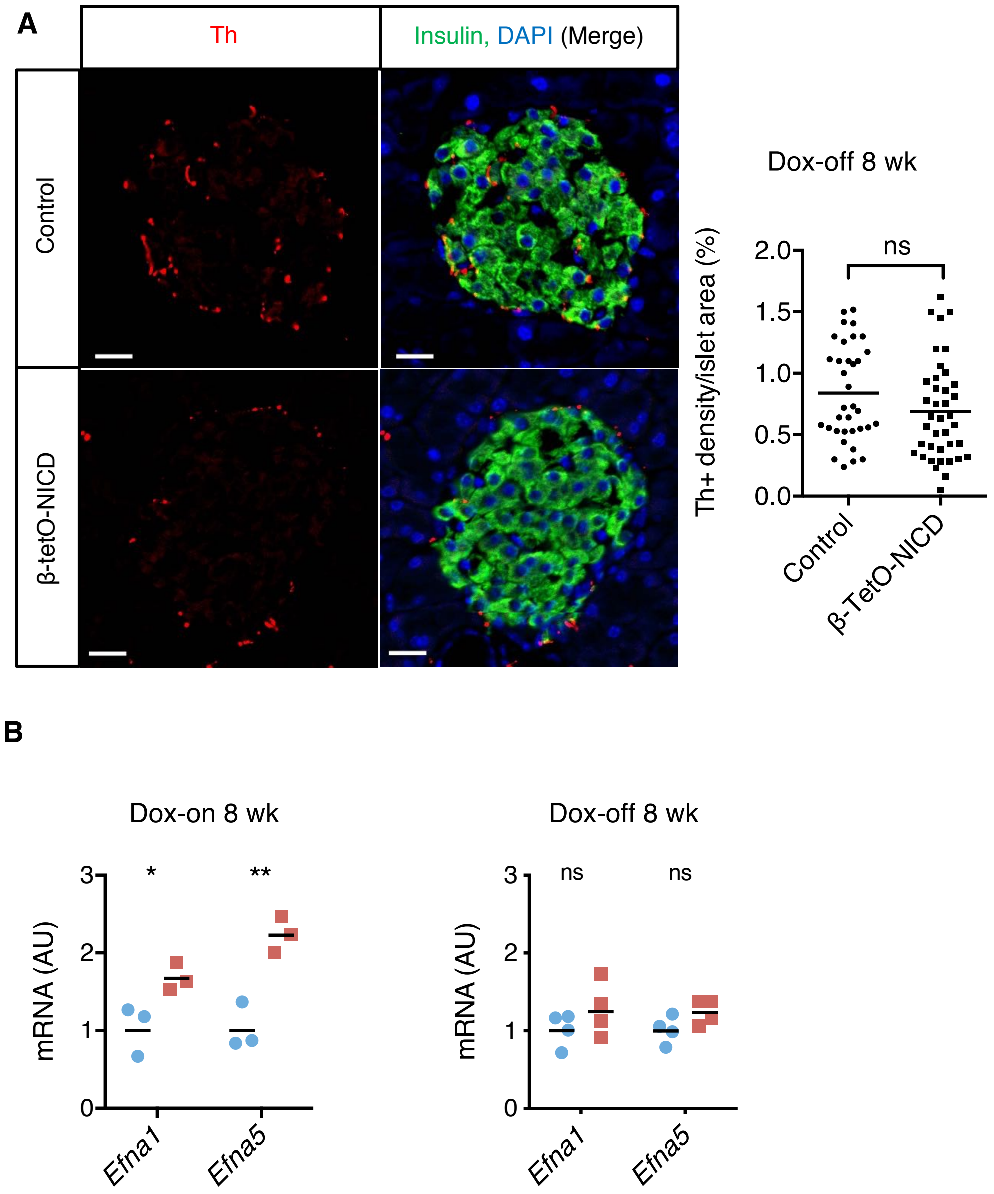


Supplemental Figure S7. Efna5 expression and islet innervation is dependent on continued Notch activity.

(A) Representative images and morphometric analysis of tyrosine hydroxylase (Th) positive area in Dox-off β-tetO-NICD and *Cre*$^-$ controls. Individual islets from at least 5 mice/group are plotted. Scale bars: 20 µm

(B) Ephrin ligand expression in islets isolated from β-tetO-NICD and *Cre*$^-$ mice after 8 weeks Dox (left) or 8 weeks Dox-off (right, N=4 mice/group).

All data are shown with group means; *, $P < 0.05$, **, $P < 0.01$ by two-tailed $t$ test.

**Supplemental Figure S8**

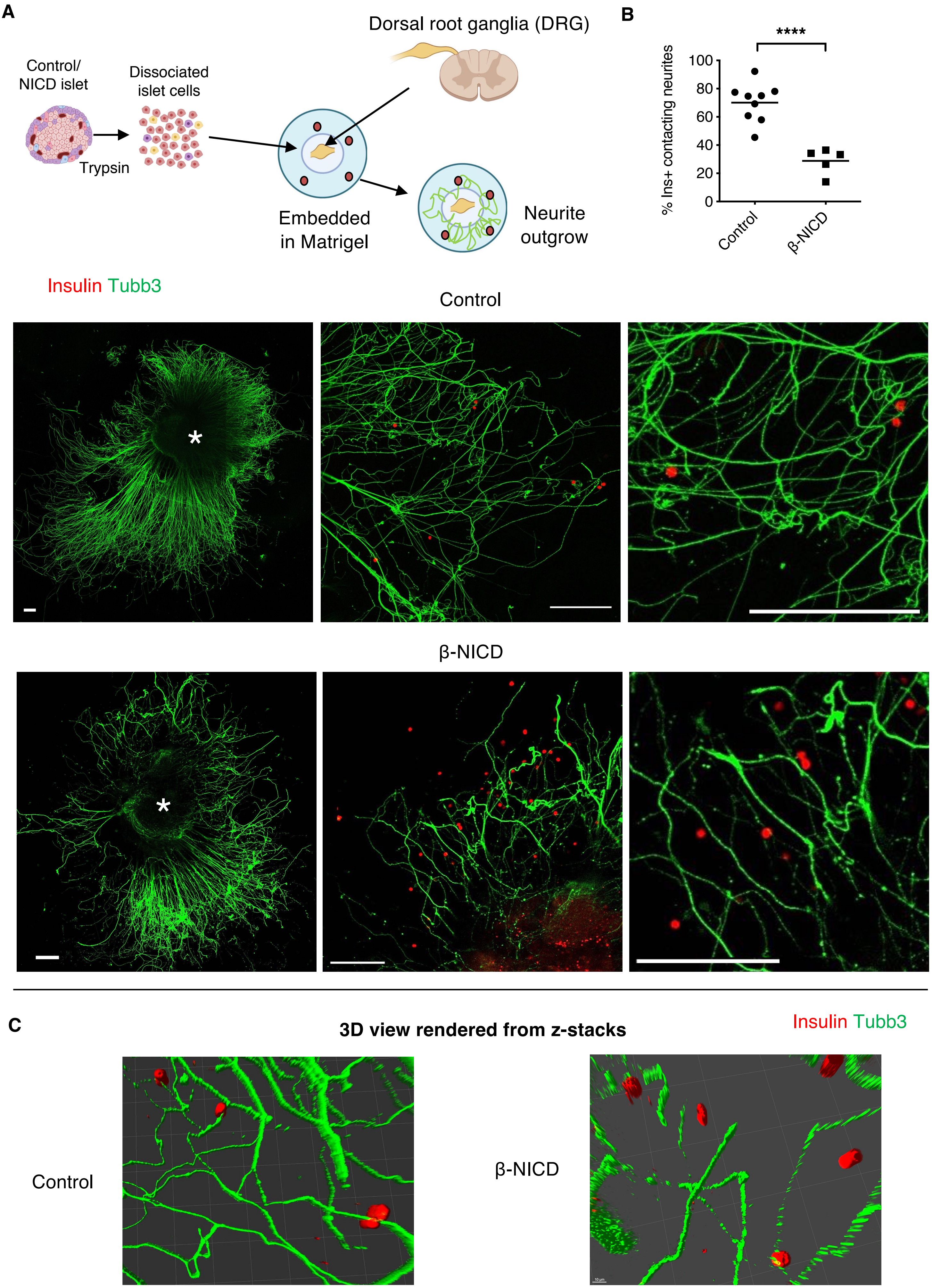

Supplemental Figure S8. Reduced neurite-β cell contact in Notch active β cells.
(A) Dorsal root ganglia (DRG) from WT mice were embedded in a Matrigel drop. Islet cells were dispersed and seeded in a Matrigel disk around the DRG explant, then cultured with nerve growth factor for one week.
(B) Representative images in cells fixed and stained with insulin (Ins) and Tubb3, and quantification on Ins+ cells contacting neurites. Scale bars: 200 µm.
(C) Three-dimensional reconstruction from z-stacks of control and β-NICD mice.
All data are shown with group means, ****, $P < 0.0001$ by two-tailed *t* test.

**Supplemental Figure S9**

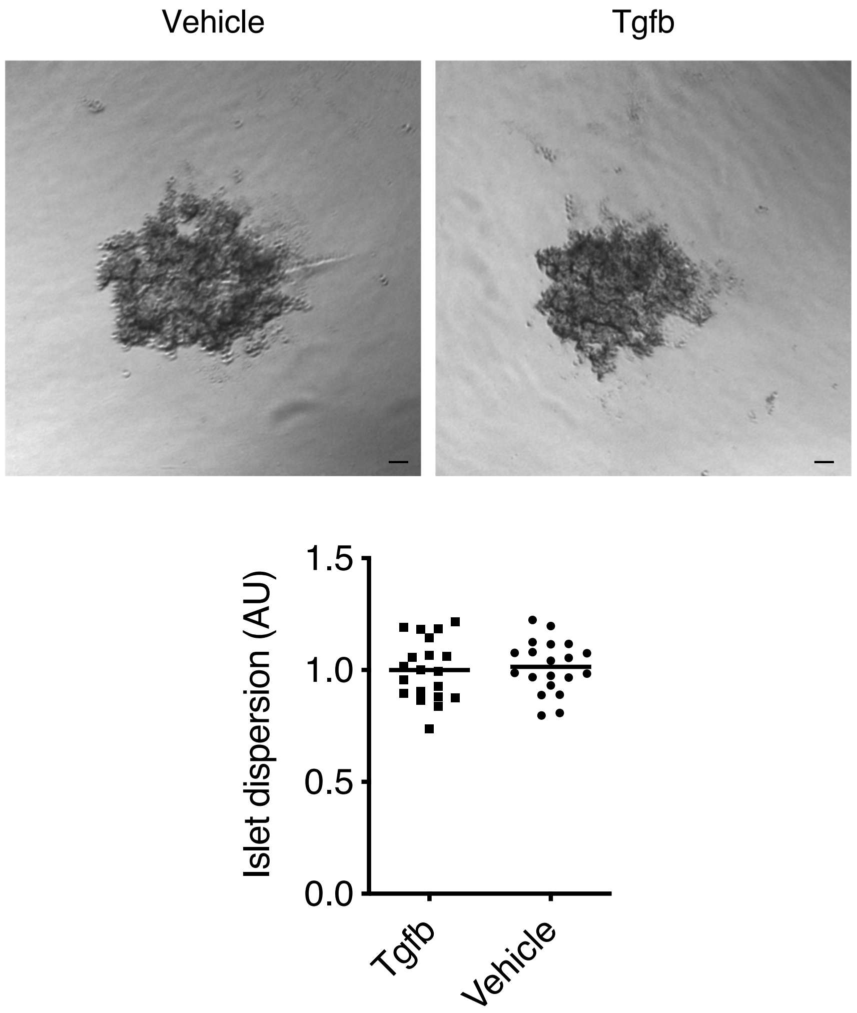


Supplemental Figure S9. TGFβ does not affect islet dispersion. Representative image and quantitation of pseudoislet formation dispersed islet cells from WT mice, in the presence of recombinant 10 ng/ml TGFβ or vehicle. Data plotted from individual pseudoislets, pooled from 3 independent experiments. All data are shown with group means. Scale bars: 20 µm

**Supplemental Figure S10**

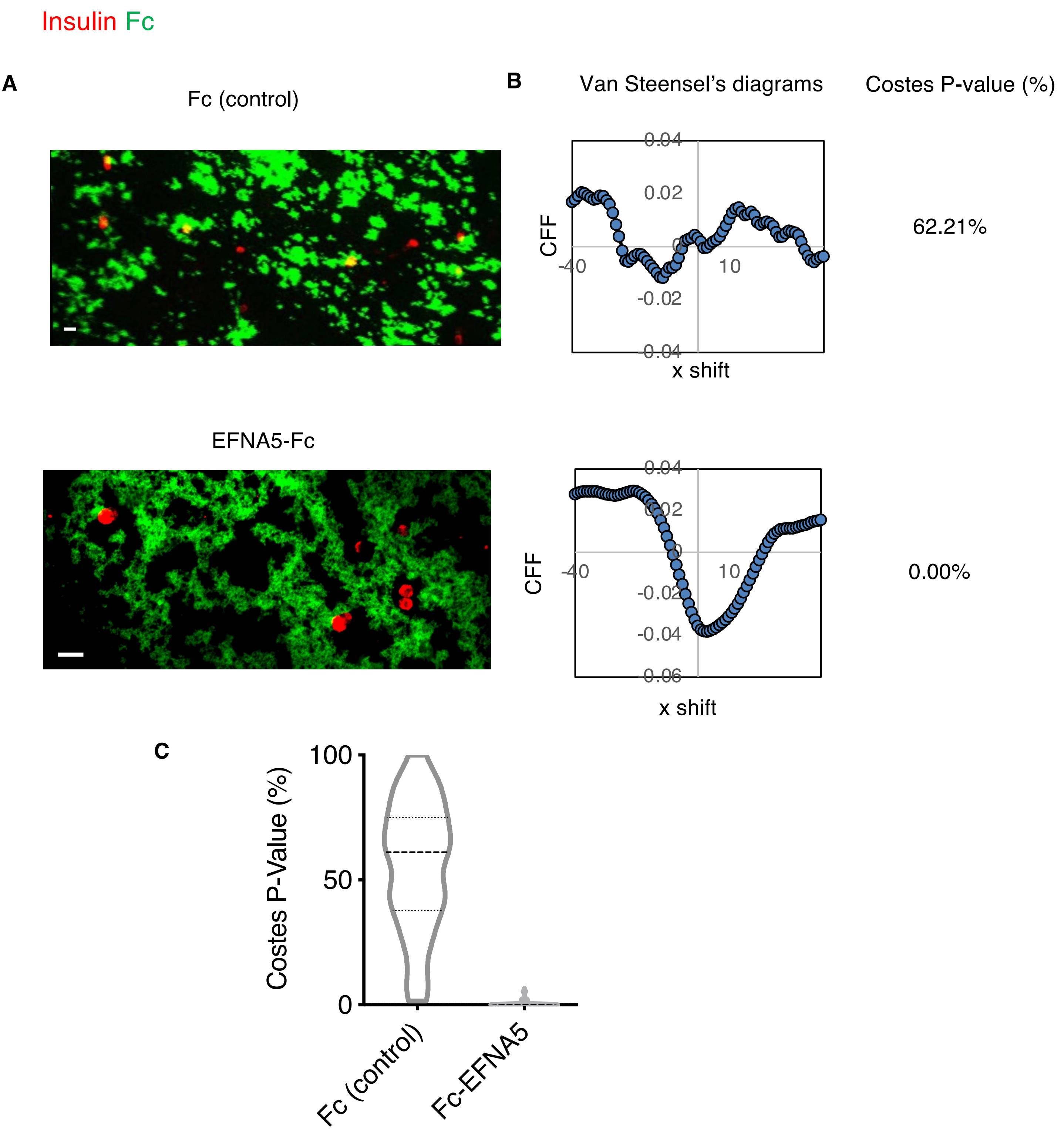


Supplemental Figure S10. Image analysis algorithms used for cell adhesion assays.
(A) Representative images of dispersed β cells seeded onto Fc (top) or EFNA5-Fc-coated surfaces (bottom), showing insulin and Fc staining. Scale bars: 20 µm.
(B) Van Steensel's cross correlation function (CFF) for cell adhesion assays. The diagram represents the variation in the correlation of green/red pixels as a function of the lateral shift of the green channel. A dip in x=0 indicates perfect anti-correlation with EFNA5-Fc (bottom graph), while cells adhered to Fc show random distribution.
(C) Distribution of Costes P-value (%) for all images analyzed.

# Supplemental Figure S11

**A**

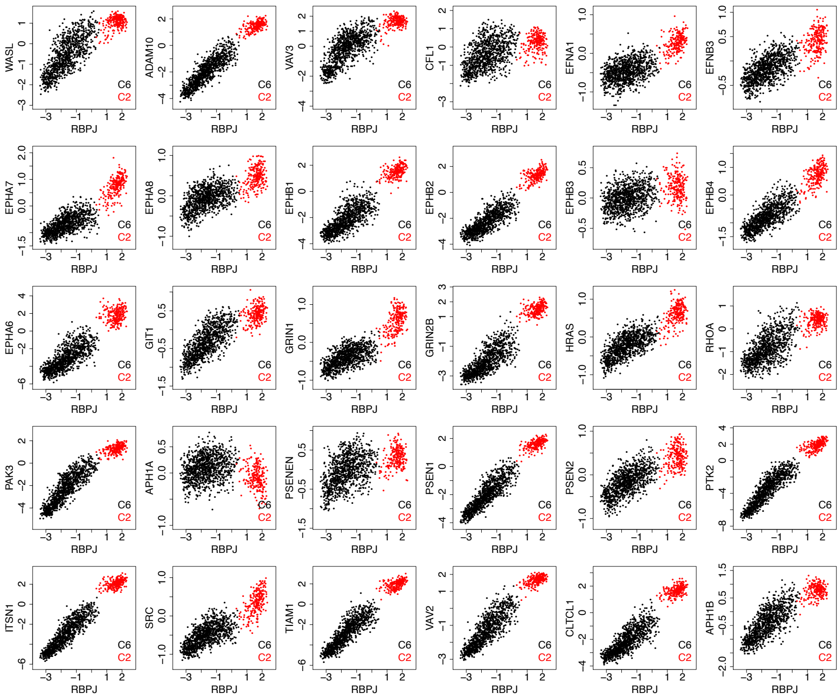


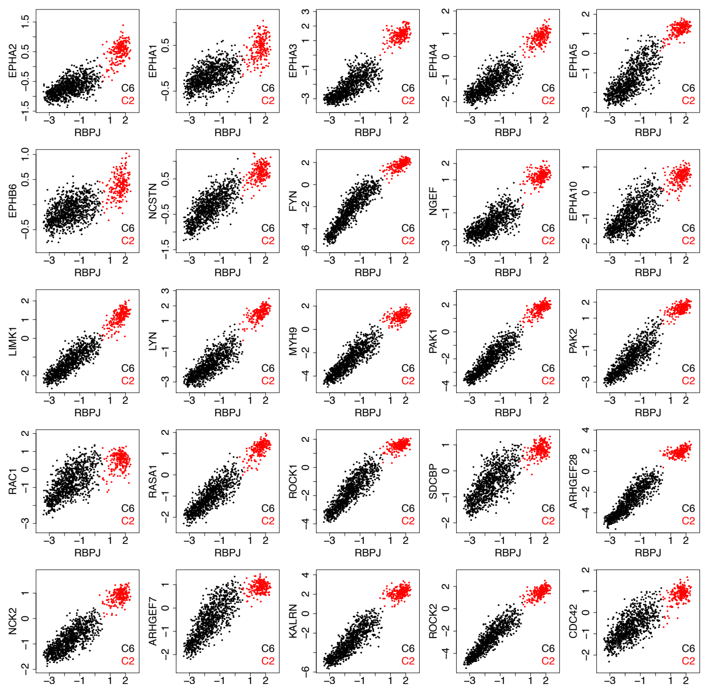


Pearson's *r* values

| | Hβ | MIβ | | Hβ | MIβ |
|---|---|---|---|---|---|
| WASL | 0.75 | 0.37 | LIMK1 | 0.83 | 0.66 |
| ADAM10 | 0.87 | 0.57 | LYN | 0.79 | 0.63 |
| VAV3 | 0.72 | 0.31 | MYH9 | 0.87 | 0.43 |
| CFL1 | 0.51 | 0.19 | PAK1 | 0.87 | 0.66 |
| EFNA1 | 0.39 | 0.41 | PAK2 | 0.84 | 0.55 |
| EFNB3 | 0.59 | 0.32 | PAK3 | 0.87 | 0.47 |
| EPHA2 | 0.57 | 0.48 | APH1A | 0.25 | -0.29 |
| EPHA1 | 0.53 | 0.33 | PSENEN | 0.47 | 0.25 |
| EPHA3 | 0.76 | 0.46 | PSEN1 | 0.88 | 0.6 |
| EPHA4 | 0.72 | 0.57 | PSEN2 | 0.64 | 0.2 |
| EPHA5 | 0.75 | 0.59 | PTK2 | 0.89 | 0.56 |
| EPHA7 | 0.64 | 0.6 | RAC1 | 0.65 | 0.24 |
| EPHA8 | 0.51 | 0.32 | RASA1 | 0.82 | 0.65 |
| EPHB1 | 0.74 | 0.42 | ROCK1 | 0.87 | 0.47 |
| EPHB2 | 0.8 | 0.52 | SDCBP | 0.71 | 0.4 |
| EPHB3 | 0.31 | -0.12 | ARHGEF28 | 0.88 | 0.47 |
| EPHB4 | 0.74 | 0.54 | ITSN1 | 0.89 | 0.44 |
| EPHB6 | 0.45 | 0.38 | SRC | 0.64 | 0.57 |
| NCSTN | 0.73 | 0.39 | TIAM1 | 0.88 | 0.46 |
| FYN | 0.9 | 0.64 | VAV2 | 0.88 | 0.53 |
| NGEF | 0.7 | 0.36 | CLRCL1 | 0.81 | 0.44 |
| EPHA10 | 0.69 | 0.4 | APH1B | 0.75 | 0.25 |
| EPHA6 | 0.75 | 0.12 | NCK2 | 0.76 | 0.42 |
| GIT1 | 0.75 | 0.29 | ARHGEF7 | 0.79 | 0.18 |
| GRIN1 | 0.59 | 0.52 | KALRN | 0.86 | 0.36 |
| GRIN2B | 0.77 | 0.36 | ROCK2 | 0.89 | 0.6 |
| KRAS | 0.66 | 0.46 | CDC42 | 0.68 | 0.44 |
| RHOA | 0.62 | 0.24 | | | |

**B**

| SYMBOL | RANK IN GENE LIST | RANK METRIC SCORE | RUNNING ES | CORE ENRICHMENT |
|---|---|---|---|---|
| EFNA5 | 39 | 0.441 | 0.0614 | Yes |
| EPHA6 | 356 | 0.346 | 0.0973 | Yes |
| EPHA3 | 378 | 0.343 | 0.1455 | Yes |
| VAV2 | 393 | 0.341 | 0.1937 | Yes |
| TIAM1 | 731 | 0.307 | 0.223 | Yes |
| AP2A1 | 1084 | 0.281 | 0.248 | Yes |
| CLTCL1 | 1100 | 0.28 | 0.2874 | Yes |
| AP2B1 | 1348 | 0.267 | 0.315 | Yes |
| PSEN1 | 1467 | 0.262 | 0.3473 | Yes |
| EPHB2 | 1630 | 0.254 | 0.3767 | Yes |
| AP2A2 | 1839 | 0.246 | 0.4029 | Yes |
| FYN | 2274 | 0.23 | 0.4171 | Yes |
| LYN | 2388 | 0.227 | 0.4447 | Yes |
| EPHB4 | 2400 | 0.227 | 0.4768 | Yes |
| DNM1 | 2692 | 0.219 | 0.4955 | Yes |
| ADAM10 | 4596 | 0.178 | 0.4384 | No |
| EPHB1 | 4702 | 0.176 | 0.459 | No |
| CLTC | 5163 | 0.167 | 0.463 | No |
| YES1 | 6180 | 0.152 | 0.4407 | No |
| EPHA4 | 6414 | 0.148 | 0.4518 | No |
| EPHA7 | 6584 | 0.146 | 0.4654 | No |
| EFNA2 | 7938 | 0.127 | 0.4248 | No |
| VAV3 | 9632 | 0.105 | 0.3664 | No |
| EPHB6 | 10161 | 0.099 | 0.3578 | No |
| SRC | 10714 | 0.093 | 0.3472 | No |
| PSEN2 | 11618 | 0.084 | 0.32 | No |
| EPHA10 | 11789 | 0.082 | 0.3244 | No |
| EPHA2 | 12414 | 0.076 | 0.3082 | No |
| NCSTN | 12816 | 0.072 | 0.301 | No |
| RAC1 | 13638 | 0.063 | 0.2744 | No |
| EPHA5 | 13644 | 0.063 | 0.2832 | No |
| EFNA1 | 13652 | 0.063 | 0.2919 | No |
| APH1A | 13809 | 0.061 | 0.2939 | No |
| EPHA1 | 13932 | 0.06 | 0.2972 | No |
| ACTG1 | 14369 | 0.056 | 0.2863 | No |
| EFNB2 | 14503 | 0.054 | 0.2882 | No |
| CLTB | 15452 | 0.045 | 0.2535 | No |
| MMP2 | 15555 | 0.044 | 0.2553 | No |
| EPHB3 | 15952 | 0.04 | 0.2438 | No |
| EFNA3 | 16059 | 0.039 | 0.2447 | No |
| APH1B | 16167 | 0.038 | 0.2455 | No |
| CLTA | 16211 | 0.037 | 0.2489 | No |
| EFNB3 | 16308 | 0.036 | 0.25 | No |
| AP2S1 | 20228 | -0.01 | 0.0814 | No |
| EFNB1 | 20468 | -0.013 | 0.073 | No |
| EFNA4 | 20953 | -0.02 | 0.0548 | No |
| MMP9 | 21038 | -0.021 | 0.0542 | No |
| ACTB | 21877 | -0.042 | 0.024 | No |
| AP2M1 | 21935 | -0.045 | 0.0279 | No |
| EPHA8 | 22178 | -0.057 | 0.0256 | No |
| PSENEN | 22671 | -0.1 | 0.0186 | No |

Supplemental Figure S11. Notch-Ephrin signaling in scRNA-seq experiments.
(A) RBPJ activity as compared to EPH-Ephrin signaling components, with corresponding Pearson's *r* values. Each dot represents a single Hβ (black) and MIβ (red) cell.
(B) List of all EPH-Ephrin gene-set components enriched in ranked MIβ vs Hβ DEGs, corresponding to GSEA plot shown in Figure 7C.

Supplemental Figure S12

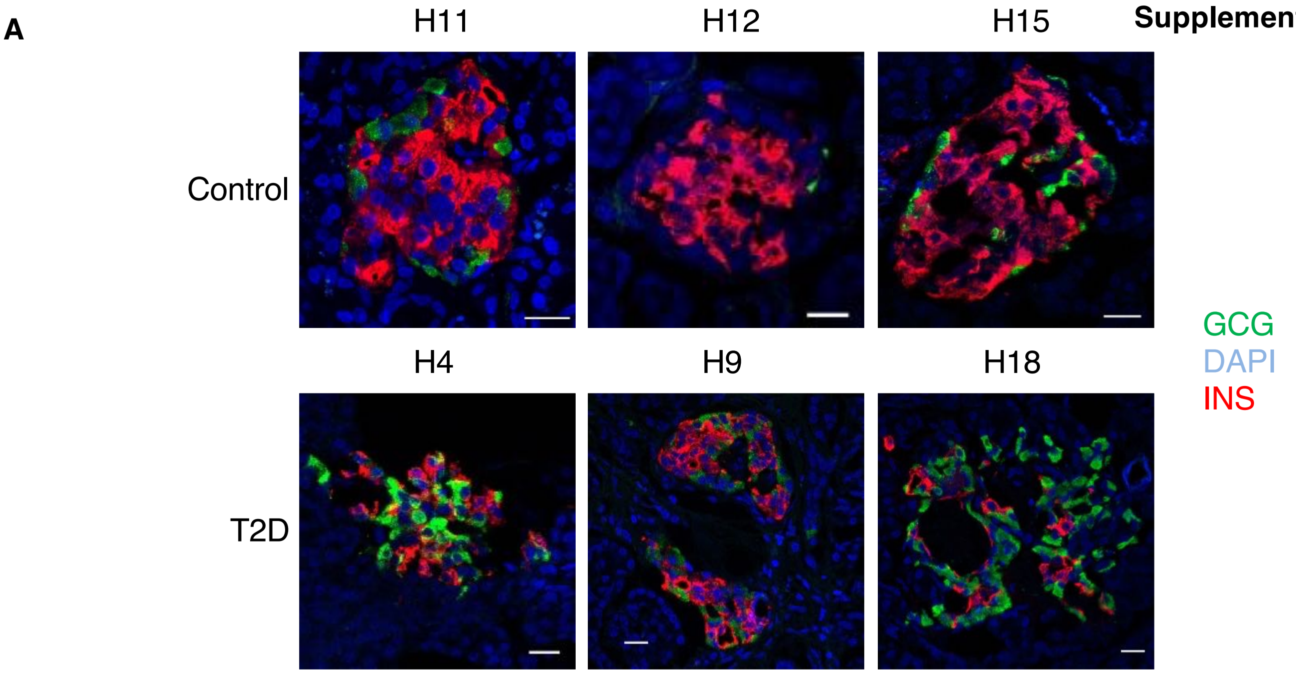


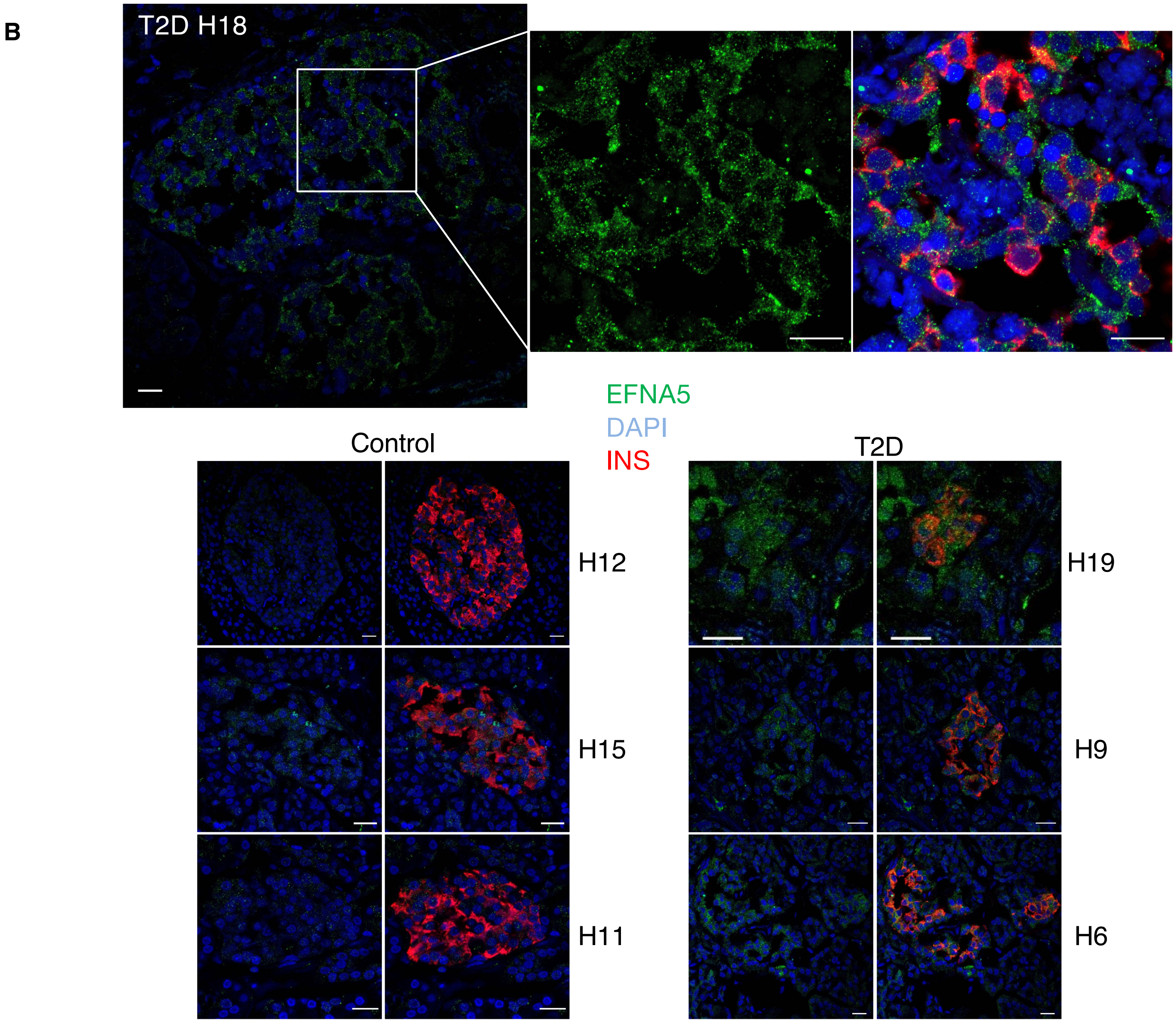


Supplemental Figure S12. Further characterization of nondiabetic and T2D patient islets.
(A) Representative Glucagon (GCG), and (B) EFNA staining (with insulin, INS) of human pancreas in nondiabetic and T2D patient islets. Scale bars: 20 µm
